# Loss of natural resistance to schistosome in T cell deficient rat

**DOI:** 10.1371/journal.pntd.0008909

**Published:** 2020-12-21

**Authors:** Liaoxun Lu, Junjian Hu, Tianzhu Chao, Zhijun Chen, Zhuangzhuang Liu, Xinsong Luo, Yinming Liang, Pei He, Lichen Zhang

**Affiliations:** 1 Laboratory of Genetic Regulators in the Immune System, Henan Collaborative Innovation Center of Molecular Diagnosis and Laboratory Medicine, Xinxiang Medical University, Xinxiang, Henan Province, China; 2 Laboratory of Mouse Genetics, Institute of Psychiatry and Neuroscience, Xinxiang Medical University, Xinxiang, Henan Province, China; 3 Hunan Institute of Parasitic Diseases, Yueyang, Hunan Province, China; 4 Henan Key Laboratory of Immunology and Targeted Therapy, School of Laboratory Medicine, Xinxiang Medical University, Xinxiang, Henan Province, China; Universidade Federal de Minas Gerais, BRAZIL

## Abstract

Schistosomiasis is among the major neglected tropical diseases and effective prevention by boosting the immune system is still not available. T cells are key cellular components governing adaptive immune response to various infections. While common laboratory mice, such as C57BL/6, are highly susceptible to schistosomiasis, the SD rats are extremely resistant. However, whether adaptive immunity is necessary for such natural resistance to schistosomiasis in rats remains to be determined. Therefore, it is necessary to establish genetic model deficient in T cells and adaptive immunity on the resistant SD background, and to characterize liver pathology during schistosomiasis. In this study we compared experimental schistosomiasis in highly susceptible C57BL/6 (B6) mice and in resistant SD rats, using cercariae of *Schistosoma japonicum*. We observed a marked T cell expansion in the spleen of infected B6 mice, but not resistant SD rats. Interestingly, *CD3e*^*−/−*^ B6 mice in which T cells are completely absent, the infectious burden of adult worms was significantly higher than that in WT mice, suggesting an anti-parasitic role for T cells in B6 mice during schistosome infection. In further experiments, we established Lck deficient SD rats by using CRISPR/Cas9 in which T cell development was completely abolished. Strikingly, we found that such Lck deficiency in SD rats severely impaired their natural resistance to schistosome infection, and fostered parasite growth. Together with an additional genetic model deficient in T cells, the *CD3e*^*−/−*^ SD rats, we confirmed the absence of T cell resulted in loss of natural resistance to schistosome infection, but also mitigated liver immunopathology. Our further experiments showed that regulatory T cell differentiation in infected SD rats was significantly decreased during schistosomiasis, in contrast to significant increase of regulatory T cells in infected B6 mice. These data suggest that T cell mediated immune tolerance facilitates persistent infection in mice but not in SD rats. The demonstration of an important role for T cells in natural resistance of SD rats to schistosomiasis provides experimental evidences supporting the rationale to boost T cell responses in humans to prevent and treat schistosomiasis.

## Author summary

Schistosomiasis is among the major neglected tropical diseases and affects mainly the developing countries. Although the role of the immune system in driving immunopathology in schistosomiasis has been extensively studied, how adaptive immunity contributes to disease resistance during schistosome infection is still not completely understood. Most livestock species as well as humans are susceptible to schistosomiasis, while some mammals are extremely resistant. The common laboratory C57BL/6 mice are highly susceptible to schistosomiasis; however, the SD rats are extremely resistant. In this study, we first used T cell deficient *CD3e*^*−/−*^ C57BL/6 mice and experimental *Schistosoma japonicum* infection and further established novel T cell deficient models in SD rats to assess anti-parasite roles of T cells. Strikingly, we found that the natural resistance of SD rat to schistosomiasis was abolished in the absence of T cells, despite the fact that the liver pathology was mitigated following infection. Therefore, our study presented experimental support for the rationale to boost T cell function for clearance of schistosome parasites.

## Introduction

Schistosomiasis is a chronic infectious disease that is pandemic in many tropical and sub-tropical regions. Epidemiological surveys in the last decades have found that human, livestock species, as well as wild rodent species are susceptible to the parasitic worms such as *Schistosoma japonicum* in Asia. However, some mammalian species are resistant to schistosomiasis, as documented in earlier studies including the laboratory SD rats, an outbred strain of *Rattus norvegicus* [1–3]. Previous studies already revealed differences regarding immunity between mouse and rat during schistosomiasis, which were related to mast cells and platelet mediated cytotoxicity [4–6]. In addition, there are numerous studies in rodent models that focus on immunopathological roles of both adaptive and innate immune cells in schistosomiasis [7,8], however far less is known about the contribution of specific immune cells to parasite clearance, and especially natural resistance to schistosomiasis in non-classical models remain poorly understood. One of the major reasons for lack of studies into natural resistance mechanisms to schistosomiasis using genetically engineered non-classical laboratory animals such as SD rats was that genetically targeting a specific cell type was not feasible before advent of highly efficient genome editing tools. Although schistosomiasis is known as consequence of parasite evasion from host immune system damage, the contribution of adaptive immunity to natural resistance in experimental models remain to be determined [9]. Recent clinical advances in immunotherapy, preventing T cell exhaustion, boosting T cell activation or releasing T cell tolerance mechanisms, have led to striking clinical benefits in cancer treatment, demonstrating that T cells can be manipulated to respond under an immunosuppressive environment and provide protective immunity, which also suggests that T cells could be harnessed to treat persistent infections [10–12]. In previous studies, T cell differentiation in mice were found to be significantly modulated during schistosomiasis [13], and T cells are involved in immunopathology including liver fibrosis[14]. However, infection experiments still lack in determining whether T cells could impact worm development and parasite load in the naturally resistant hosts, which is at least partially due to difficulties to obtain appropriate genetic models. For example, in the SD rats it remains unknown whether deficiency in T cells could impact its natural resistance to schistosome infection such as *S*. *japonicum* which induces severe pathology in B6 mice [15]. Additionally, *S*. *japonicum* infection does induce immune tolerance via differentiation of regulatory T (Treg) cells which express the master transcription factor Foxp3, it is not known whether Treg cell versus effector T cell differentiation in rats is implicated in resistant phenotype in SD rats [16]. Due to the recent development of genetic engineering approaches in oocytes using CRISPR/Cas9 mediated gene editing, it is now more feasible to generate genetically modified non-conventional laboratory animal strains including rats. This offers the opportunity to explore the roles of specific components of immune system in natural resistance to schistosomiasis using genetic models. In this study, we first used *CD3e*^*−/−*^ B6 mice deficient in T cells to analyze consequences of T cell deficiency in animals infected by *S*. *japonicum* cercariae [17]. More importantly, we obtained a novel rat genetic model, by inactivating the essential T cell molecule Lck to completely remove functional T cells in SD rats, using CRISPR/Cas9 genome editing. With these genetic models deficient in T cell development in both the susceptible B6 mice and resistant SD rats, we found that T cells significantly restrict schistosomiasis in the susceptible B6 mice, and strikingly, Lck deficiency in SD rats severely impaired natural resistance to *S*. *japonicum* infection.

In an additional genetic model deficient in T cells, the *CD3e*^*−/−*^ SD rats, we confirmed T cell absence caused loss of natural resistance to schistosome infection but mitigated immunopathology. Such experiments using genetic models determined the decisive role of T cells in natural resistance of SD rats which therefore provided mechanistic insights into prevention of schistosomiasis.

## Materials and methods

### Ethics statement

All animal studies were performed under the guidelines approved by the Animal Ethics Committee of Xinxiang Medical University (China).

### Animals

C57BL/6 mice and SD rats were purchased from Beijing Vital River Laboratory Animal Technology (China). *CD3e*^*−/−*^ mice were kindly provided by Dr. Bernard Malissen from France (Centre d’Immunologie de Marseille-Luminy) and bred in our Specific Pathogen-Free facility.

### Generation and genotyping *Lck*^−/−^ SD rats and *CD3e*^*−/−*^ SD rats

Two single-guide RNA or sgRNAs located in exon 3 and exon 4 of rat *Lck* gene were designed using the online CRISPOR tool (http://crispor.tefor.net/). Cas9 mRNA and the sgRNAs were prepared as previously described [18]. CRISPR/Cas9 microinjection and transplant of rat embryos were conducted as the standard protocol [19]. The tail tips from F0 pups were used for isolation of genomic DNA. CRISPR/Cas9 targeted region of *Lck* was amplified with standard PCR and subcloning of PCR products were subjected for Sanger sequencing for identification of mutated alleles. Genotyping primers of rat *Lck* gene: sense 5’-ATTTCCTACCCCACAGCTGC-3’, and antisense, 5’-CCCCGTATCAAGCACTCTGG-3’. Additional genetic model of T cell deficient on SD rat background was obtained using identical protocol as used for *Lck* knockout in SD rats. *Cd3e* targeting using CRISPR/Cas9 in SD rats was shown in S7 Fig. Genotyping primers of *Cd3e* gene in SD rats: sense 5’-GACTTTTCCCCTCCCACCTC-3’, and antisense 5’- TGGTCAGTGTTGGTCATCAGAG-3’.

### Schistosoma japonicum infection

*Oncomelania hupensis* snail infected with *S*. *japonicum*, were obtained from an endemic area in Hunan Province, China. *S*. *japonicum* cercariae were collected using a needle from the water surface after being shed from infected snails exposed for 3~4 h to artificial light. Mice were infected with 20 cercariae, SD rats with 100 cercariae per individual, respectively.

### Parasite analysis

Infected animals were sacrificed and dissected 42 days post-infection. Parasite were perfused through portal and mesenteric veins [20]. Worms were transferred into petri dish and washed with saline. The collected worms were counted and photographed using Nikon SMZ745T microscope with Digital Sight DS-Fi2 camera. Worm width and length were determined based on digital micrographs using NIS-Elements D 4.60.00 software. Worm recovery rate in each group was calculated from the number of worms perfused at 6 weeks post-infection versus the number of cercariae infected initially.

### Flow cytometry and cell sorting

Immunophenotyping was performed in mice or rats in steady state or after infection. Peripheral blood cells, splenocytes, lymph nodes and thymocytes were stained with monoclonal antibody mixes and analyzed by flow cytometry. Cells were first counted by using Invitrogen Attune NxT Flow Cytometer (Thermo Fisher Scientific) and then 1 million cells were stained in 100 μl of staining buffer with antibody mixes, and acquired on the FACSCanto flow cytometry system (BD Biosciences, USA). The FACS data was analyzed using FlowJo software version 10.0. Splenic CD4^+^ T cells from mouse or rat were sorted by BD FACSAria Fusion. To avoid T cell activation mediated downregulation of CD3, we used CD5 and CD4 to gate on CD4^+^ T cells in mice [21]. For each cell sorting experiments, BD FACS Accudrop Beads (BD Biosciences, USA; Cat No.: 345249) were used to calibrate the instrument for to ensure sorting accuracy. We selected “purity” mode for each sorting experiment.

### Real-time quantitative PCR

Total RNA was extracted from sorted splenic CD4^+^ T cells using RNeasy Plus Micro Kit (Qiagen, Cat No./ID: 74034) and from liver of mice or rat using RNeasy Plus Mini Kit (Qiagen, Cat No./ID: 74134). For sorted cells 20~60 ng of total RNA was used to synthesize cDNA with Random Primer Mix (New England Biolabs) in 20 μl reactions using SuperScript IV Reverse Transcriptase (Thermo Fisher Scientific). cDNA samples were diluted 1:5 and 2 μl used in 20 μl RT-qPCR reactions together with 10 μl SYBY Select Master Mix (Thermo Fisher Scientific). Real-time PCR assays were done on a 7500/7500 Fast Real-Time PCR System (Applied Biosystems, Thermo Fisher Scientific). Relative gene expression was calculated by the 2^(−ΔCT)^ method using different housekeeping gene controls, *Hprt* gene for mice and *Sdha* gene for rat [22]. Gene-specific primers are listed in S1 Table.

### Enzyme linked immunosorbent assay

Blood samples were collected from mice and rats with *S*. *japonicum* infection of 42 days. The sandwich enzyme-linked immunosorbent assay (ELISA) were used to detect specific IgG anti-*S*. *japonicum* soluble worm antigen preparation (SWAP) specific IgG antibodies in sera from mice (Shanghai Jianglai Biotech, China; Cat No.: JL49959-96T) and rats (Shanghai Jianglai Biotech, China; Cat No.:JL49955-96T) respectively, according to the assay kit manufacturer’s instructions. The absorbance values were measured at 450 nm in a microplate reader (GloMax Discover and Explorer System, Promega). ELISA assays were performed in triplicate and the background signals were subtracted. Standard curves were produced using standards provided in the ELISA kits and employed to calculate serum concentration of schistosome-specific IgG.

### Pathologic analyses of the liver

Liver samples were immediately fixed with 4% paraformaldehyde and then embedded in paraffin. For histological analysis, liver sections (5 μm) were stained with H&E to assess liver granulomas and Masson’s trichrome to assess liver fibrosis. For Immunohistochemical analysis, CD11b and CD19 staining were performed according to standard procedures. In briefly, sections were incubated with CD11b (Servicebio, China; Cat No.: GB11058) or CD19 (Servicebio, China; Cat No.: GB11061-1) overnight and the next day stained with secondary antibody. The nucleus was stained with hematoxylin and then treated with diaminobenzidine. Images were acquired on a Pannoramic MIDI II (3D HISTECH). The positive areas were measured by using CaseViewer software (3D HISTECH).

### Statistical analysis

All data were analyzed with GraphPad Prism software (version 8.0). Data are presented as means ± s.e.m. Statistical significance was assessed by parametric (unpaired Student’s *t* test) and nonparametric (Mann–Whitney test) methods when two groups were compared. *P* values less than 0.05 were considered significant (*, *P*<0.05; **, *P*<0.01; ***, *P*<0.001; ****, *P*<0.0001), NS, non-significant.

## Results

### Worm load and liver pathology in T cell deficient *CD3e*^*−/−*^ C57BL/6 mice following *S*. *japonicum* infection

**Fig 1:**
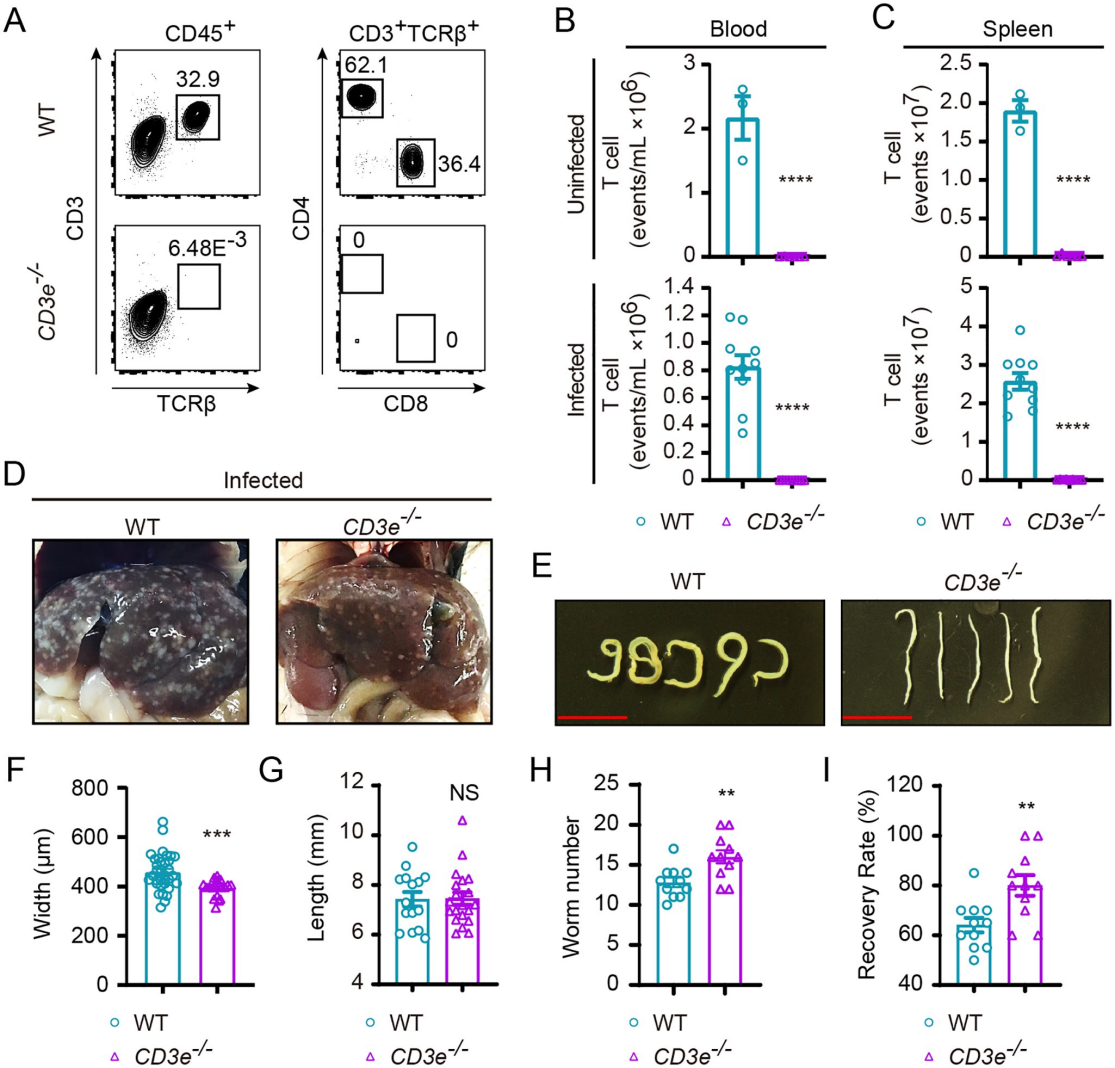
T cell phenotyping and parasite load in WT B6 and *CD3e*^*−/−*^ mouse following *S*. *japonicum* infection. (A) Representative FACS gating of immune cells in spleen from wild-type (WT) B6 and *CD3e*^*−/−*^ mice. Expression of CD4 and CD8 by T cells (CD45^+^CD3^+^TCRβ^+^; identified at left) from the blood of WT B6 (top) and *CD3e*^*−/−*^ mice (bottom). Numbers adjacent to outlined areas indicate percentage of cells in each gate. (B, C) Total numbers of T cells of blood (B) or spleen samples (C) from *CD3e*^*−/−*^ mice compared with those from WT B6 mice uninfected (top) or 6 weeks post-infection (bottom). Data of infected mice are from two independent experiments (uninfected condition: WT, n = 3; *CD3e*^*−/−*^, n = 6, infected: WT, n = 10; *CD3e*^*−/−*^, n = 9). (D) Comparison of liver pathological changes in B6 (left) and *CD3e*^*−/−*^ mouse (right) at 6 weeks post-infection. (E) Representative micrographs of worms harvested from portal and mesenteric veins of infected B6 mouse (top) and *CD3e*^*−/−*^ mice (bottom) 6 weeks after cercariae infection (Scale bar, 5 mm.). (F) Width of worms collected from portal and mesenteric veins of WT and *CD3e*^*−/−*^ mouse 6-week after infection was determined from digital micrographs. Data are from three experiments (WT, n = 34; *CD3e*^*−/−*^, n = 19). (G) Length of worms recovered from WT and *CD3e*^*−/−*^ mice at 6-week post-challenge was measured based on digital micrographs (WT, n = 15; *CD3e*^*−/−*^, n = 20). (H) Number of worms recovered from WT and *CD3e*^*−/−*^ mice at 6 weeks after challenge (WT, n = 11; *CD3e*^*−/−*^, n = 11). (I) The recovery rate of *S*. *japonicum* collected from portal and mesenteric veins of infected B6 mouse and *CD3e*^*−/−*^ mouse. Data were from three independent experiments (WT, n = 11; *CD3e*^*−/−*^, n = 11). The following fluorochrome-tagged antibodies were used for FACS analyses: anti-mouse CD45-APC-eFluor 780, anti-mouse CD4-PE-Cyanine7, anti-mouse CD3ε-PE-Cyanine5.5, anti-mouse TCR beta-FITC, anti-mouse CD8α -Alexa Fluor 700. Data represent the mean ± s.e.m. Statistical significance was assessed by unpaired Student’s *t*-test or non-parametric unpaired Mann-Whitney test and indicated by * *P*<0.05, **** *P*<0.0001, NS, non-significant.

*S*. *japonicum* infects a large diversity of mammals including humans and most of livestock [23]. Immunopathological alterations following infection affect liver, lungs and gastrointestinal tracks that could result in organ failure and death [24,25]. Even though well documented in previous studies that T lymphocytes or T cells are essential for development of liver pathology when C57BL/6 or B6 and BALB/c mice were infected by *S*. *mansoni* [26], the role of T cells in pathology during *S*. *japonicum* infection remains to be elucidated in T cell deficient genetic models. We adopted a protocol to challenge adult B6 mice with 20 cercariae of *S*. *japonicum* via skin infection with cover-glass aided exposure of the abdominal region of mice following fur removal [27]. By 42 d following percutaneous infection, we observed in C57BL/6 mice grossly visible liver granuloma, significantly increased CD4 and CD8 T cell numbers in spleen, regardless of the significant drop of CD4 T cells in peripheral blood (S1 Fig). In further experiments, we sought to analyze T cell deficient mice with *Cd3e* gene knockout on C57BL/6 background during *S*. *japonicum* infection. CD3e is essential for the T cell receptor complex, loss of which could completely abolish T cell development, therefore differing from Rag recombinase deficient mice lacking both T and B cells [17]. *CD3e*^*−/−*^ B6 mice were completely absent in T cells, and such a model permits this study to analyze in a more specific manner how T cells are involved in host defense against the highly infectious parasite species *S*. *japonicum*. To further explore the role of T cells during *S*. *japonicum* infection, we compared *CD3e*^*−/−*^ B6 mice deficient in T cell to WT B6 controls for worm growth and worm load 42 d following cercarial challenge. In both steady state and *S*. *japonicum* infected mice, *CD3e*^*−/−*^ B6 mice were found completely deprived of peripheral T cells in blood and spleen (Fig 1A, 1B and 1C). As T cells participate in tissue damage during infection [14], it was not surprising for us to find that the liver granuloma of *CD3e*^*−/−*^ B6 mice which were deprived of T cells was visible but apparently alleviated when it was compared to granuloma in infected WT control mice (Fig 1D). However, it is interesting to note that the worm development was significantly affected by CD3e deficiency, with 1.2-fold decrease in worm width which was quantitated based on digital micrographs, while the length of the worm recovered from WT mice and CD3e deficient mice exhibited no significant differences (Fig 1E, 1F and 1G). Interestingly, despite impeded development of adult worms which was in line with previous studies using nude mice [28], additionally our experiments showed that both worm count and recovery rate of worm in *CD3e*^*−/−*^ B6 mice was significantly higher than those in WT mice at 42 d after infection (Fig 1H and 1I, and S2 Fig).

We further compared liver pathology between wild-type and *CD3e*^*−/−*^ B6 mice by analyzing granuloma and fibrosis using H&E staining and Masson’s trichrome staining, as well as fibrosis related marker genes expression. In *CD3e*^*−/−*^ B6 mice, the granuloma formation, as well as scaring severity were significantly inhibited (S3A–S3D Fig). Induction of fibrosis related marker genes was also significantly reduced in the T cell deficient mice during *S*. *japonicum* infection (S3E Fig). We also compared CD11b^+^ myeloid cells and B lymphocytes in *CD3e*^*−/−*^ B6 mice and wild-type mice following infection, and found that accumulation of such immune cells in liver was markedly decreased when T cells were absent (S3F and S3G Fig). Therefore, our findings revealed that T cell deficiency in CD3e knockout mice causes worm-load increase but inhibited liver fibrosis.

### Resistance of SD rats to schistosome infection and characteristics of their T cells

**Fig 2:**
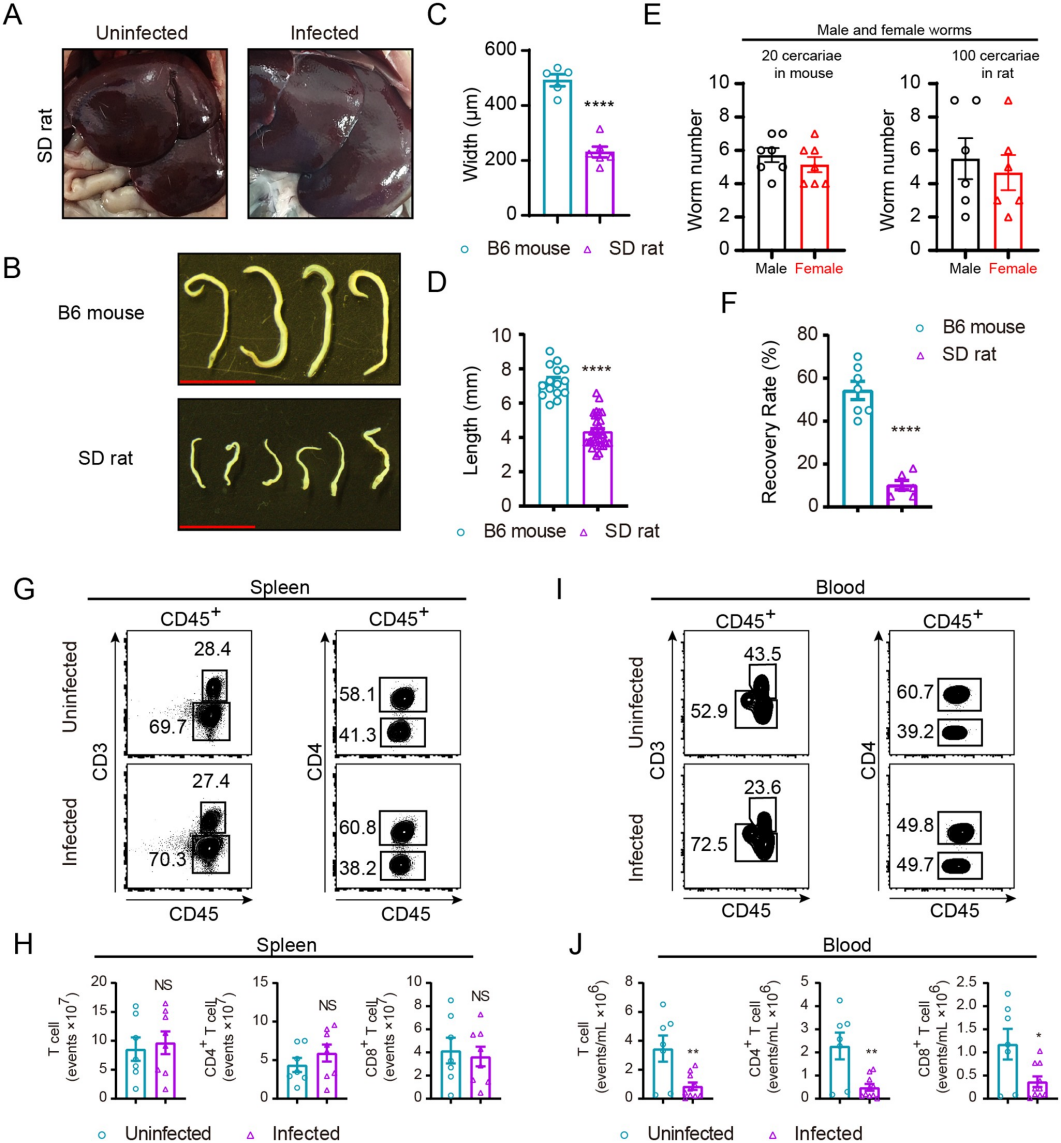
Immunopathologic characteristics of SD rat after *S*. *japonicum* infection. (A) *S*. *japonicum* infection does not cause overt liver granuloma in SD rat, liver of uninfected SD rat (left) and liver of infected (right). (B) Representative micrographs of worms collected from portal and mesenteric veins of B6 mouse (left) and SD rat (right) both infected with *S*. *japonicum* for 6 weeks (Scale bar, 5 mm). (C) Width of worms from B6 mice and SD rats at week 6 post-challenge was measured based on digital micrographs. Data are from two experiments (B6 mouse, n = 5; SD rat, n = 6). (D) Length of worms harvested from portal and mesenteric veins of infected B6 mouse and SD rat was determined. Data are from two independent experiments (B6 mouse, n = 15; SD rat, n = 33). (E) Number of male and female worms recovered from B6 mouse and SD rat at 6-week post-challenge. (F) Percentage of worm recovery from portal and mesenteric veins of infected B6 mouse and SD rat, presented from two experiments (B6 mouse, n = 7; SD rat, n = 6). (G-I) Representative FACS plots of immune cells in spleen (G) or peripheral blood (I) of SD rat under uninfected and infected conditions. (H, J) Absolute number of total T cells, CD4^+^ or CD8^+^ T cells in G and I. Data are from two independent experiments (H, uninfected, n = 7; infected, n = 8. J, uninfected, n = 7; infected, n = 10). The following fluorochrome-tagged antibodies were used: anti-rat CD45 -eFluor450, anti-rat CD3-APC, anti-rat CD4-APC/Cyanine7, anti-rat CD8α-PE. Data represent the mean ± s.e.m. Statistical significance was assessed by unpaired Student’s *t*-test or non-parametric unpaired Mann-Whitney test and indicated by * *P*<0.05, ** *P*<0.01, **** *P*<0.0001, NS, non-significant.

In order to reveal the involvement and importance of T cells in *S*. *japonicum* infection, we further performed comparative study in the resistant SD rats. As documented in previous studies SD rats are outbred animals which are naturally resistant to *S*. *japonicum* infection in contrast to the B6 mice [2,29]. First, we found that no granuloma was grossly visible for the liver from infected rats, dramatically different from the infected B6 mice (Fig 2A). We also found that infection with 100 cercariae of *S*. *japonicum* resulted in 10% worm recovery in rat in contrast to that of 54% in infected mice with 20 cercariae, and the worms collected from SD rats were noticeably smaller than those from B6 mice (Fig 2B). When quantitated by width and length, the worms collected from SD rats were 2.1-fold and 1.7-fold smaller in width and length respectively than those of worms from B6 mice following 42 d of infection (Fig 2C and 2D). Our experiments did confirm that SD rats are far more resistant to *S*. *japonicum* infection than B6 mice as shown by marked differences in worm numbers and recovery rates (Fig 2E and 2F and S4 Fig).

In further experiments, we analyzed T cells following *S*. *japonicum* infection in SD rats. Interestingly, in splenocytes of infected SD rats we observed no significant changes in total T cells or in CD4, CD8 subsets following infection in terms of cell count (Fig 2G and 2H). Such results were different from the increase of T cells in spleen of infected B6 mice. In addition, in peripheral blood of infected SD rats the absolute counts of total T cells as well as the CD4, CD8 subsets of T cells were significantly decreased in comparison to those of uninfected SD rats (Fig 2I and 2J). Such data showed that SD rats were notably resistant to *S*. *japonicum* infection, as the parasite load was significantly lower than that in B6 mice, and there was significant impediment of worm development in wild-type SD rats. In addition, T cell characteristics displayed significant differences between the susceptible mice and resistant SD rats. Therefore, it is intriguing to analyze such natural resistance to schistosomiasis in the absence of T cells using a genetic rat model, to determine the T cell roles in controlling *S*. *japonicum* infection.

### Generation of T cell deficient rats on the resistant SD background by Lck deletion

**Fig 3:**
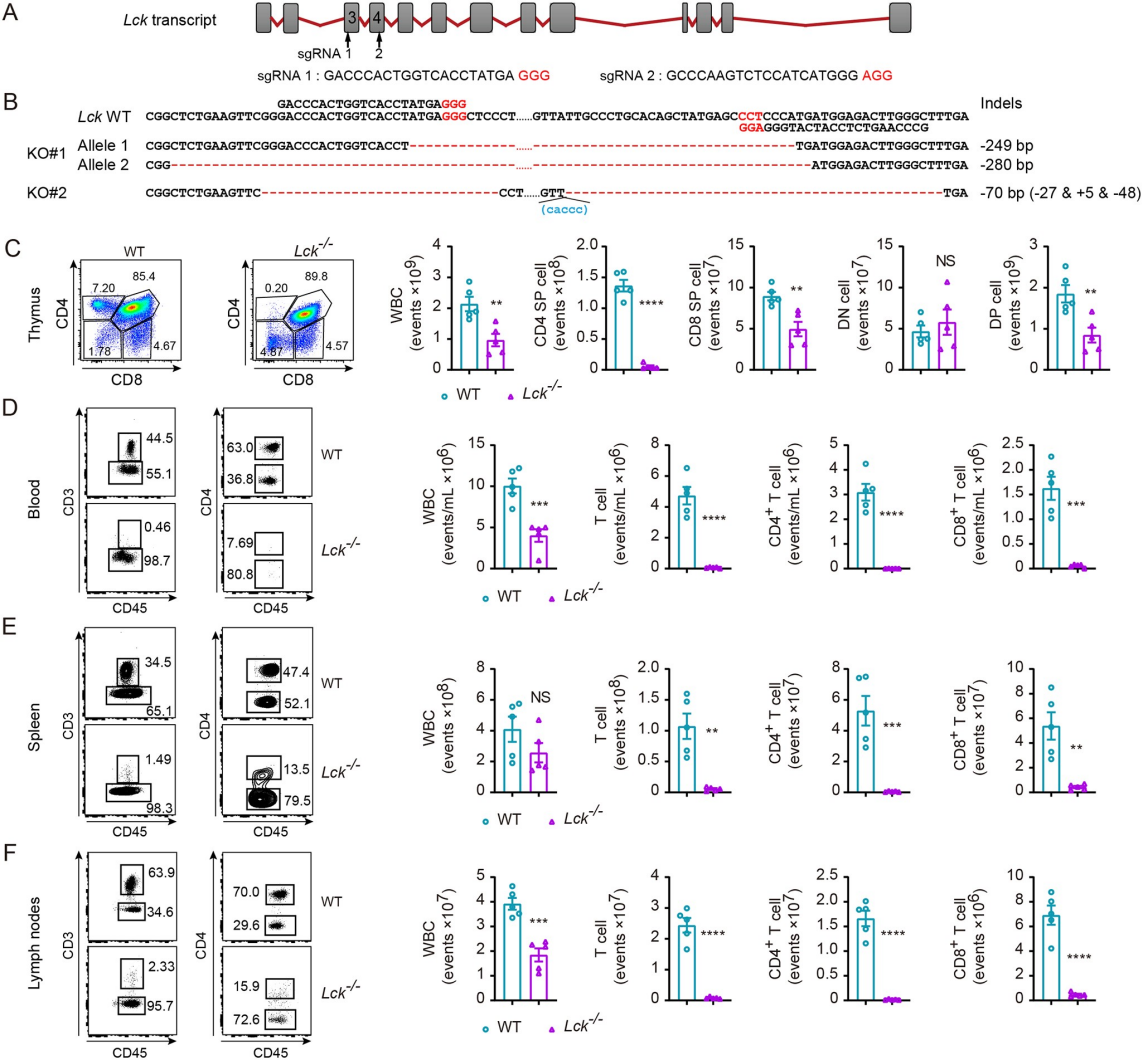
Generation and analysis of *Lck* knock-out SD rat. (A) Schematic diagram of the rat *Lck* locus and sgRNA targeting sequences. sgRNA 1 sequence located in exon 3 and sgRNA 2 in exon 4 were indicated respectively. (B) DNA sequencing analysis of mutant alleles from two *Lck* mutant founders (KO#1 and KO#2) confirms genome editing. Sanger sequencing results showed large-fragment deletions indicated in red dashes. Sequences non shown between sgRNA 1 and sgRNA 2 were as indicated in black dots. The protospacer adjacent motifs (PAM) were indicated in red letters in A or B. (C) Expression of CD4 and CD8 T cells by WT rat and *Lck*^*−/−*^ rat thymocytes (left), analyzed by flow cytometry. Absolute number of white blood cell (WBC), CD4^+^ or CD8^+^ single positive (SP) cells, double-negative (DN) CD4^−^CD8^−^ thymocytes and double-positive (DP) CD4^+^CD8^+^ thymocytes (right). (D, E, F) Representative flow cytometry of lymphocytes in peripheral blood (D), spleen (E), or lymph nodes (F) of WT SD rat and *Lck*^*−/−*^ SD rat (left). Numbers adjacent to outlined areas (left) indicate percent cells in each gate; absolute number of WBC, CD3^+^, CD4^+^ or CD8^+^ T cells in blood (D), spleen (E), or lymph nodes (F) of WT SD rat and *Lck*^*−/−*^ SD rat (right). C−F, numbers adjacent to outlined areas indicate percentage of each population (left); data from two independent experiments (n = 5 per group). The following fluorochrome-tagged antibodies were used: anti-rat CD45-eFluor450, anti-rat CD3-APC, anti-rat CD4-APC/Cyanine7, anti-rat CD8α-PE. Data represent the mean ± s.e.m. Statistical significance was assessed by unpaired Student’s *t*-test or non-parametric unpaired Mann-Whitney test and indicated by ** *P*<0.01, *** *P*<0.001, **** *P*<0.0001, NS, non-significant.

To determine the contribution of T cells in SD rats to their natural resistance to *S*. *japonicum* infection, we applied CRISPR/Cas9 genome editing tool to genetically inactivate *Lck* gene which is essential for TCR signaling and T cell development [30,31]. *Lck* is a highly specific T cell gene and the Cre recombinase driven under Lck promoter has been frequently used to perform gene deletion selectively in T cells [32,33]. In our experiments, two independent sgRNAs were designed to target exon 3 and exon 4 of Lck (Fig 3A). Sequencing validation was performed both in F0 founder rats (Fig 3B) and the progeny, which were obtained following backcrossing the founder to wild-type SD rats and intercrossing the heterozygotes of *Lck* mutated F1 animals (data not shown). By staining T cells with anti-rat CD45, CD3, CD4, CD8 monoclonal antibodies, we found that homozygous rats with *Lck* deletion were absent in T cells in peripheral blood, spleen, lymph nodes. In thymocytes we found that in *Lck*^*−/−*^ SD rats T cell development was severely impaired in both double positive (DP) and single positive (SP) stages while we did not find difference in cell number in the double negative stage (Fig 3C). It is of note that the CD4^+^ SP cells in thymus were dramatically decreased in mutant rats, and the CD8^+^ SP cells were decreased to a less severe extend. As immature CD8 single positive thymocytes giving rise to DP cells, fall into the CD8^+^ compartment, we postulate that Lck deficiency in rats has more dramatic impact on mature SP cells. Consistently, we found CD3^+^ T cells were absent in peripheral blood, spleen and lymph nodes (Fig 3D–3F). Our observation concerning T cell development in Lck deficient rats was in line with Lck deficient mice which had a dramatic reduction in the DP thymocytes, and undetectable mature SP thymocytes, accompanied by absence of peripheral T cells [34]. Therefore, we obtained by CRISPR/Cas9 genome editing tool a novel genetic rat model deficient in T cells, which could be used to determine T cell role in natural resistance of SD rats to *S*. *japonicum* infection.

### Loss of resistance to *S*. *japonicum* infection in T cell deficient *Lck*^*−/−*^ SD rats

**Fig 4:**
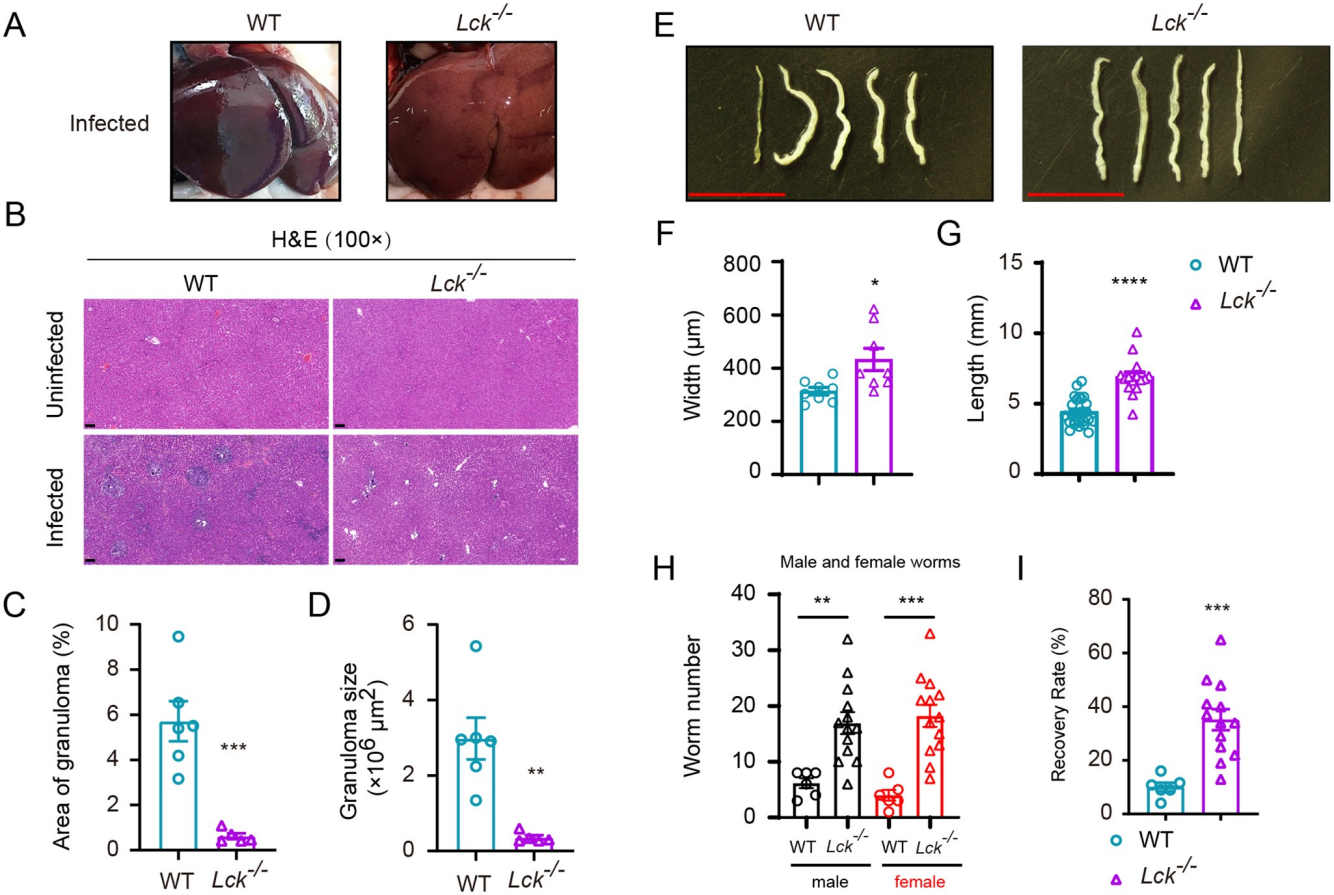
Immunopathologic analysis of *Lck*^*−/−*^ SD rat following schistosome infection. (A) *S*. *japonicum* skin infection does not cause noticeable liver granuloma in *Lck*^*−/−*^ SD rat (right) compared to the liver of infected WT rat control (left). (B) H&E staining of liver sections from uninfected or infected WT SD rat and *Lck*^*−/−*^ SD rat. (Original magnification, ×100; Scale bar, 100 μm). (C, D) Granuloma size was determined based on H&E staining of liver sections using CaseViewer software (WT rat, n = 6; *Lck*^*−/−*^ rat, n = 5). (E) Representative micrographs of *S*. *japonicum* recovered from portal and mesenteric veins of infected WT SD rat (left) or *Lck*^*−/−*^ SD rat (right) at 42 days after infection (Scale bar, 5 mm). (F) Width of worms from WT rat and *Lck*^*−/−*^ rat at week 6 post-challenge was measured based on digital micrographs (worms from WT rats, n=8; worms from *Lck*^*−/−*^ rat, n = 8). (D) Length of worms harvested from infected WT SD rat and *Lck*^*−/−*^ SD rat. Data are from two independent experiments (worms from WT rat, n = 25; worms from *Lck*^*−/−*^ rat, n = 15). (H) Number of male and female worms recovered from WT and *Lck*^*−/−*^ SD rat at 6-week post-infection. (I) The recovery rate of *S*. *japonicum* collected from infected WT rat and *Lck*^*−/−*^ SD rat. Data are from two independent experiments (WT rat, n = 6; *Lck*^*−/−*^ SD rat, n = 13). Data represent the mean ± s.e.m. Statistical significance was assessed by unpaired Student’s *t*-test or non-parametric unpaired Mann-Whitney test and indicated by * *P*<0.05, ** *P*<0.01, *** *P*<0.001, **** *P*<0.0001.

To analyze resistance of SD rats to *S*. *japonicum* infection in the absence of T cells, we infected *Lck*^*−/−*^ SD rats and wild-type controls 100 cercariae per animal and calculated worm recovery by 42 d post-infection. As shown in Fig 4A, liver granuloma was not grossly noticeable in both *Lck*^*−/−*^ SD rats and wild-type controls 42 d after infection. However, in H&E staining the liver sections from *Lck*^*−/−*^ SD rats displayed significantly less schistosome egg-induced hepatic granuloma (Fig 4B–4D). Surprisingly, we found that the worm size in T cell deficient rats was enlarged when they were compared to worms collected from wild-type rats, which was the reverse of the results found in T cell deficient B6 mice. Worm width and length were significantly increased in worms collected from T cell deficient *Lck*^*−/−*^ SD rats, which was opposite to the results observed in T cell deficient mice. The width and length of the worm collected from mutant rats were 1.4- and 1.6-fold increased, receptively (Fig 4E–4G). More strikingly, the number of worms collected from *Lck*^*−/−*^ SD rats was significantly higher than that from the wild-type controls, both of which were infected with 100 cercariae of *S*. *japonicum* (Fig 4H and S5 Fig), and the recovery rate of adult worms from T cell deficient *Lck*^*−/−*^ SD rats increased to 35.2 percent which was 3.5-fold higher than that of T cell sufficient SD control rats (Fig 4I). The data from infection experiments in genetically engineered SD rats provided evidences to support that T cells are essential to maintain the natural resistance of SD rats to *S*. *japonicum* infection. Loss of T cells rendered in large the loss of natural resistance of SD rats to *S*. *japonicum* infection, and absence of T cells facilitated growth of adult parasites contradicting the findings from mouse experiments in which *CD3e*^*−/−*^ mouse had smaller adult worms found at perfusion.

### Mitigated liver fibrosis in T cell deficient SD rats following *S*. *japonicum* infection

**Fig 5:**
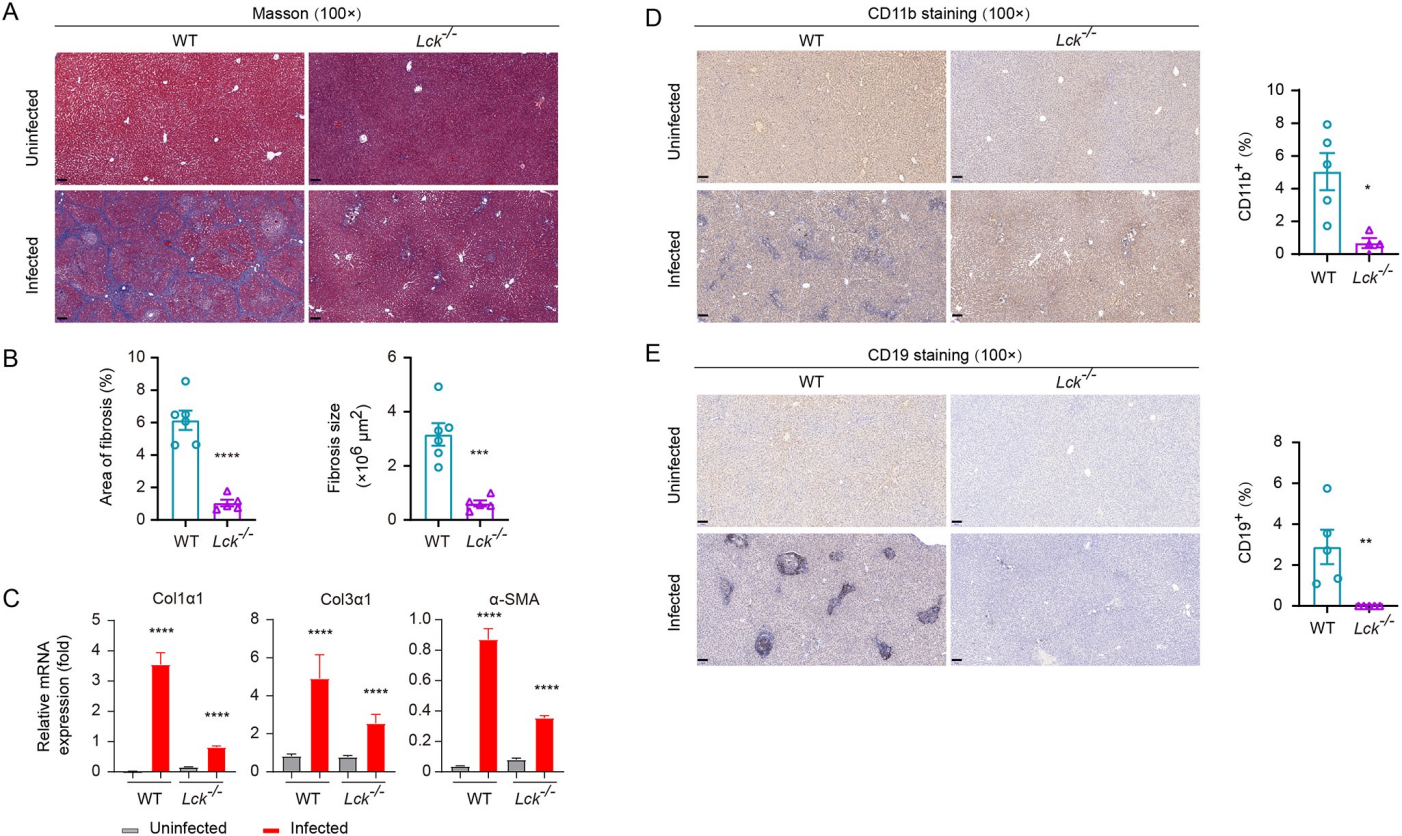
Pathologic analysis of WT and *Lck*^*−/−*^ rat after *S*. *japonicum* infection. (A) Masson’s trichrome staining of liver sections from uninfected or infected WT and *Lck*^−/−^ rat. (Original magnification, ×100; Scale bar, 100 μm). (B) Fibrotic areas in Masson’s trichrome staining of liver sections using CaseViewer software (WT, n = 6; *Lck*^−/−^, n = 5). (C) qPCR analysis of mRNA expression for fibrosis-related genes in liver of uninfected or infected WT and *Lck*^−/−^ rats: *Col1α1*, *Col3α1* and *α-SMA*. (D) CD11b staining of liver sections from uninfected or infected WT and *Lck*^−/−^ rat (left); CD11b^+^ area shown as percentage measured from CD11b stained liver sections using CaseViewer software (right) (WT, n = 5; *Lck*^−/−^, n = 5). Original magnification, ×100; Scale bar, 100 μm. (E) CD19 staining of liver sections from uninfected or infected WT and *Lck*^−/−^ rat (left); CD19^+^ area in percentage measured by CD19 staining of liver sections using CaseViewer software (right) (WT, n = 5; *Lck*^−/−^, n = 5). Original magnification, ×100; Scale bar, 100 μm. Statistical significance was assessed by unpaired Student’s *t*-test or non-parametric unpaired Mann-Whitney test and indicated by * *P*<0.05, ** *P*<0.01, *** *P*<0.001, **** *P*<0.0001.

Schistosomiasis is regarded as the most frequent cause of liver fibrosis as this common parasitic infection affects over 240 million people in tropical countries [35]. Since we observed that the worm load in *Lck*^*−/−*^ SD rats was significantly higher than that in wild-type controls 42 d after infection of 100 cercariae per animal, it was necessary to analyze accumulation of the other immune cells such as B lymphocytes and myeloid cells, as well as fibrosis formation in the liver of T cell deficient and sufficient hosts. Previous studies found that both lymphocytes and myeloid cells were involved in liver fibrosis during schistosomiasis and the other induced liver fibrosis models [36,37]. In our study, *Lck*^*−/−*^ SD rats were completely deprived of T cells and we analyzed their liver fibrosis in contrast to infected wild-type controls. Masson’s trichrome staining of liver sections showed that fibrosis could occur in wild-type SD rats 42 d after infection with 100 cercariae, but the severity of fibrosis was notably lower in *Lck*^*−/−*^ SD rats (Fig 5A and 5B). When quantitating the fibrosis related marker genes such as Col1a1, Col3a1 and alpha-SMA by qPCR, we found the induction of fibrosis marker genes was significantly inhibited when the T cells were absent in SD rats (Fig 5C). We further measured the accumulation CD11b^+^ myeloid cells and B lymphocytes in the liver of both wild-type and *Lck*^*−/−*^ SD rats 42 d after infection. In the absence of T cells, we found that both B lymphocytes and CD11b^+^ myeloid cells were dramatically decreased for their accumulation in the liver of rats during *S*. *japonicum* infection (Fig 5D and 5E). We further compared the fibrosis phenotype and liver accumulation of B lymphocytes and CD11b^+^ myeloid cells in an independent T cell deficient SD rat model which had *Cd3e* deletion by CRISPR/Cas9 (S6 Fig). First, our experiments found that *Cd3e*^*−/−*^ SD rats that were subject to infection of 100 cercariae per animal for 42 d, also had significantly higher load of worm than the wild-type controls (S7A–S7C Fig). Importantly, *Cd3e*^*−/−*^ SD rats also exhibited identical phenotype of liver granuloma and hepatic fibrosis as observed in the *Lck*^*−/−*^ SD rats when the infected liver sections were analyzed by H&E staining and Masson’s trichrome staining, as well as by qPCR analyses of fibrosis related marker genes (S7D–S7G Fig). T cell deficiency in *Cd3e*^*−/−*^ SD rats had dramatic reduction in liver accumulation of CD11b^+^ myeloid cells and B lymphocytes (S7H and S7I Fig). We also tested the serum antibody level in T cell deficient mice and rats after infection, and as expected the IgG levels in both T cells deficient mice and rats were significantly reduced (S8 Fig). In our study we confirmed that loss of T cells in both *Lck*^*−/−*^ SD rats and *Cd3e*^*−/−*^ SD rats, liver fibrosis was significantly diminished accompanied by dramatic reduction CD11b^+^ myeloid cells and B lymphocytes in the infected liver, suggesting that even in the resistant SD rats T cells are involved in both resistance to infection and also play important role in development of liver fibrosis.

### Regulatory T cells could be involved in robust anti-schistosomiasis T cell response in SD rats

**Fig 6:**
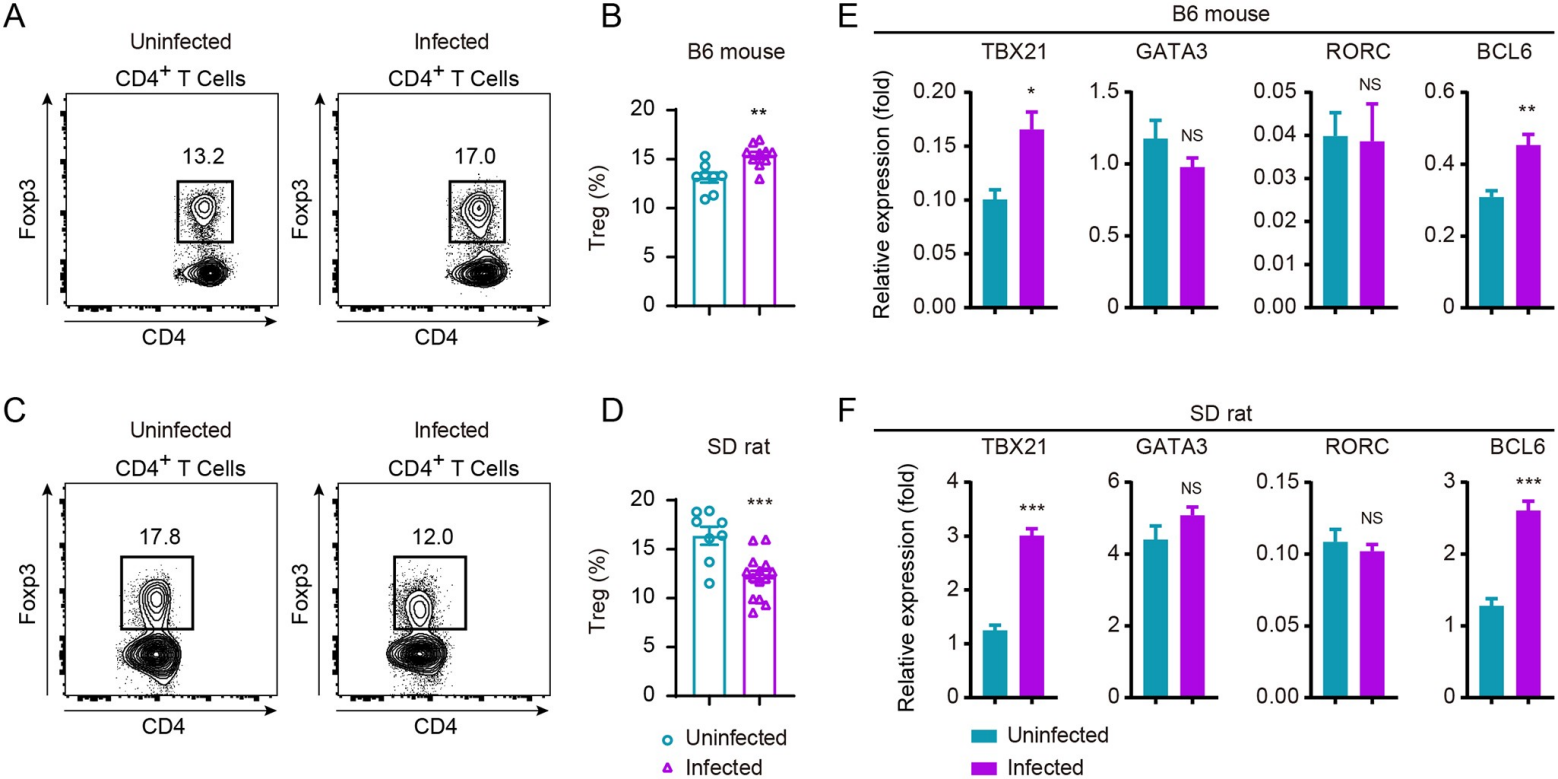
Comparison of Treg frequency and transcription factors expression of B6 mouse and SD rat upon *S*. *japonicum* infection. (A, C) FACS analysis of FOXP3^+^ Tregs from B6 mouse (A) or SD rat (C) under uninfected and infected conditions. Expression of FOXP3 on CD4^+^ T cells from uninfected and 6-week S. *japonicum*-infected mice or rat. (B) Quantification of data in A. Data is representative of two experiments (uninfected B6 mouse, n = 8; infected B6 mouse, n = 10). (D) Quantification of data in C. Results represent for three experiments (uninfected rat, n = 8; infected rat, n = 14). (E, F) mRNA expression of transcription factor TBX21 (T-bet), GATA3, RORC or BCL6 of FACS-sorted CD4^+^ T cells from B6 mouse (E) or SD rat (F) under uninfected and infected conditions by using real-time PCR. *Hprt* or *Sdha* was used as internal controls for mice or rat, respectively. Relative mRNA levels are presented as mean ± s.e.m. The following fluorochrome-tagged antibodies were used: anti-mouse CD45-APC-eFluor 780, anti-mouse CD5-eFluor 450, anti-mouse CD4-Brilliant Violet 605, anti-mouse FOXP3-PE, anti-rat CD45-eFluor450, anti-rat CD3-APC, anti-rat CD4-APC/Cyanine7, anti-rat FOXP3-PE. Data represent the mean ± s.e.m. Statistical significance was assessed by unpaired Student’s *t*-test or non-parametric unpaired Mann-Whitney test and indicated by *, *P*<0.05; **, *P*<0.01; ***, *P*<0.0001; NS, non-significant.

Peripheral tolerance of T cells prevents eradication of pathogens and tumors mediated by a particular subset of T cells termed regulatory T cells [38,39]. Persistent infection typically results in Treg cell differentiation which leads to immune tolerance [40–42]. In line with results from previous studies that Treg cells could be induced following schistosome infection [43], we found that Treg cells were induced during *S*. *japonicum* infection in B6 mice (Fig 6A and 6B). Treg cells were significantly increased in B6 mice following 42 d of infection, however on the contrary frequencies of Treg cells in SD rats were significantly decreased (Fig 6C and 6D), suggesting inhibition in T cell response via Treg cells occurred in mice but not in the resistant SD rats. Data from such experiments revealing differences in Treg differentiation between mice and rats during schistosome infection suggested its role in the natural resistance of SD rats against schistosomiasis. To further uncover T cell differentiation in B6 mice and SD rats following schistosome infection, we compared at mRNA level of master transcription factors involving T-bet (encoded by gene Tbx21), GATA3 (encoded by gene *Gata3*), RORγt (encoded by gene *Rorc*) and BCL6 (encoded by gene *Bcl6*), which governing Th1, Th2, Th17 and T-follicular helper (Tfh) cell differentiation, respectively. mRNA level of Tbx21 and Bcl6 were increased both in mice and rats, while Gata3 and RORγt were not found be significantly altered when we analyzed the sorted CD4^+^ T cells 42 d after infection (Fig 6E and 6F). Therefore, no significant clues were identified between B6 mice and SD rats by comparing the master transcription factors responsible for effector T cell differentiation.

## Discussion

Schistosomiasis affecting as many as 220 million people worldwide is an infectious disease by Schistosome parasites, which are mainly found in tropical regions in the Middle East, South America, Southeast Asia and sub-Saharan Africa [44,45]. The WHO has estimated more than 200,000 annual deaths related to schistosomiasis (https://www.who.int/en/news-room/fact-sheets/detail/schistosomiasis). Even though it is among the deadliest neglected tropical diseases, fundamental researches dissecting immunologic and genetic basis for disease susceptibility versus disease resistance in mammals are still limited. Like humans some rodents such as mice are highly susceptible to infection with schistosome parasites, while some rodents and primates were found to be naturally resistant [46]. Early studies using SD rats demonstrated involvement of adaptive immunity in protection against *S*. *mansoni*, and other studies showed platelet participation in inhibition of worm survival *in vitro* [5,47]. In the non-permissive water voles termed *Microtus Fortis* inhabiting the endemic lake regions, granulocytes were found to be involved in clearing parasites, and bone marrow transplant of this water vole conferred resistance phenotype in immunodeficient recipient mice following schistosome infection [48,49]. However, the specific roles of T cells in natural resistance to schistosomiasis *in vivo* have not been determined, largely due to the fact that genetic manipulation of the non-permissive animals were not feasible.

In this study, we first compared the T cell response, worm growth and liver pathology following infection with cercariae of *S*. *japonicum* in the highly susceptible C57BL/6 mice. Interestingly, in the C57BL/6 mice after 42 d of infection, T cells in peripheral blood were significantly declined but T cell number was significantly increased in spleen. Even though it is a highly susceptible strain, C57BL/6 mice still keep low level of resistance to worm growth, as *CD3e*^*−/−*^ B6 mice completely absent in T cells had significantly higher level of adult worm recovery than wild-type controls. Interestingly, the presence of T cells fosters development of adult worms, as we observed that T cell deficient CD3e knockout mice had significantly decreased width of adult worms which was in line with previous studies [50]. Consistent with previous studies that absence of T cells attenuated liver granuloma and hepatic fibrosis [28], *CD3e*^*−/−*^ B6 mice selectively deficient in T cells had dramatic decrease of myeloid cell accumulation and significantly mitigated liver granuloma and fibrosis following schistosome infection. It is important to note that the Rag1 or Rag2 deficiency, or the Foxn1 deficiency of nude mice led to absence of T cells but also affect the other cell types such as loss of B cells in Rag1/2 knockout mice, and impairment of multiple organs beyond T cell deficiency in Foxn1 deficient mice and patients [51,52].

Next, we found that no liver granuloma grossly visible in SD rats was developed following *S*. *japonicum* infection, which was in striking contrast to the severe liver granuloma of C57BL/6 mice following 42 d of infection. In such infected rats, we found that T cell count was not increased in spleen. Since T cells in B6 mice still had inhibiting function against *S*. *japonicum* infection, and T cell characteristics in infected SD rats were different from those in B6 mice, we postulated that T cells could contribute to the natural resistance of SD rats to schistosomiasis. To unambiguously determine the role of T cells in SD rats for their resistance to schistosomiasis, genetic model deficient in T cells was highly desirable. As an essential gene for T cell development, deletion of *Lck* in resistant rats by CRISPR/Cas9 could provide a T cell deficient model to analyze sequelae of infection, we performed genome editing experiments in SD rats and successfully established homozygous mutant animals that were completely and permanently deprived of T cells. Strikingly, in such T cell deficient rats infected with cercariae of *S*. *japonicum*, we found that width and length of the worm were increased in comparison to those measured in wild-type hosts. Such results were opposite to the fact that T cells in mice foster development of adult worm even though T cells in mice could still exert anti-parasitic functions. Our experiments also proved that absence of T cells in SD rats abolished its resistance to schistosomiasis as adult worm recovery increased from 10.1% in wild-type controls to 35.2% in *Lck*^*−/−*^ SD rats. In addition, we obtained another T cell deficient SD rat strain by targeting *Cd3e* with CRISPR/Cas9. In the CD3e^*−/−*^ SD rats, we found the same phenotype including loss of resistance to schistosomiasis, and mitigated liver granuloma and fibrosis. To explore the mechanisms underlying natural resistance of SD rats to schistosome infection which requires T cells, in further experiments we studied the differentiation of multiple subsets of T cells, including Treg cells and effector T cells. We found that Treg cells, which are major force of immunotolerance in periphery, displayed discrepant phenotype between B6 mice and SD rats. In mice, Treg cell frequencies increased significantly in infected mice with cercariae of *S*. *japonicum*, whereas their counterparts in SD rats decreased significantly. At mRNA level we did not find obvious differences in master transcription factors driving T cell differentiation such as T-bet, Gata3, RORγt and Bcl6, between the susceptible mice and resistant rats. Taken together, our comparative studies using mice and SD rats, and more importantly T cell deficient genetic models of mice and rats determined the critical role of T cells to maintain natural resistance to schistosomiasis in mammals. In this study, we found that T cells responded differentially to schistosome infection in that T cells did not change in count in spleen of SD rats but increased significantly in B6 mice. Absence in schistosomiasis-induced Treg differentiation in SD rats may explain partially its resistance to parasites, but additional mechanistic insights into the effector T cells for parasite rejection are also necessary for future investigation.

## Supporting information

S1 Fig T cell analyses of B6 mice at 6 weeks post-infection with *S*. *japonicum*. (A) liver granuloma of B6 mice 6 weeks after infection with 20 cercariae of *S*. *japonicum*, uninfected (left), infected (right). (B) Multiparameter FACS analysis of splenocytes in B6 mice under uninfected and infected conditions, identifying T cells (CD5^+^) and myeloid cells (CD11b^+^) among CD45^+^ white blood cells (left), and further subcategorizing CD5^+^ T cells into CD4^+^ or CD8^+^ cells (right). Numbers adjacent to outlined areas indicate percent cells in each gate. (C) Quantification of the data in (B). (D) Absolute number of total T cells, CD4^+^ or CD8^+^ T cells in spleen from uninfected and schistosome infected B6 mice. Results from two independent experiments (uninfected, n = 8; infected, n = 10). (E) Representative FACS analysis of white blood cells and T cells in peripheral blood of uninfected B6 or 6-week infected mice. (F, G) Percentages and absolute number of total T cells, CD4^+^ or CD8^+^ T cells in E. Results represented for two independent experiments (uninfected, n = 8; infected, n = 10). The following fluorochrome-tagged antibodies were used: anti-mouse CD45-APC-eFluor 780, anti-mouse CD5-PE-Cyanine7, anti-mouse CD11b APC, anti-mouse CD4-FITC, anti-mouse CD8α-Alexa Fluor 700. Data represent the mean ± s.e.m. Statistical significance was assessed by unpaired Student’s *t*-test or non-parametric unpaired Mann-Whitney test and indicated by * *P*<0.05, *** *P*<0.001, **** *P*<0.0001, NS, non-significant.
(TIF)

S2 Fig Numbers of male and female worms recovered from WT and *CD3e*^*−/−*^ mouse at 6-week post-challenge. Data are from three independent experiments (Experiment 1: WT, n = 3; *CD3e*^*−/−*^, n = 3. Experiment 2: WT, n = 3; *CD3e*^*−/−*^, n = 3. Experiment 3: WT, n = 5; *CD3e*^*−/−*^, n = 5).
(TIF)

S3 Fig Pathologic analysis of WT B6 and *CD3e*^*−/−*^ mouse 6 weeks after *S*. *japonicum* infection. (A) H&E staining of liver sections from uninfected or infected WT B6 and *CD3e*^*−/−*^ mouse. (Original magnification, ×100; Scale bar, 100 μm.). (B) Granuloma size was quantified from H&E stained liver sections using CaseViewer software (WT, n = 6; *CD3e*^*−/−*^, n = 4). (C) Masson’s trichrome staining of liver sections from uninfected or infected WT B6 and *CD3e*^*−/−*^ mouse. (Original magnification, ×100; Scale bar, 100 μm.). (D) Fibrotic areas from Masson’s trichrome staining of liver sections by using CaseViewer software (WT, n = 6; *CD3e*^*−/−*^, n = 4). (E) qPCR analysis of mRNA expression level of fibrosis-related genes: *Col1α1*, *Col3α1* and *α-SMA* in the liver tissues from uninfected or infected WT B6 and *CD3e*^*−/−*^ mouse. (F) CD11b staining of liver sections from infected or uninfected WT B6 and *CD3e*^*−/−*^ mouse. (Original magnification, ×100; Scale bar, 100 μm.) (left). CD11b^+^ area shown in percentage measured from CD11b stained liver sections by using CaseViewer software (WT, n = 5; *CD3e*^*−/−*^, n = 4) (right). (G) CD19 staining of liver sections from infected or uninfected WT B6 and *CD3e*^*−/−*^ mouse. (Original magnification, ×100; Scale bar, 100 μm.) (left). CD19^+^ area shown in percentage measured from CD19 staining of liver sections by using CaseViewer software (WT, n = 5; *CD3e*^*−/−*^, n = 4) (right). Data represent the mean ± s.e.m. Statistical significance was assessed by unpaired Student’s *t*-test or non-parametric unpaired Mann-Whitney test and indicated by * *P*<0.05, **** *P*<0.0001, NS, non-significant.
(TIF)

S4 Fig Number of worms recovered from infected mice and rats. Numbers of worms from WT B6 mouse (A) and WT SD rat (B) at 6-week post-challenge. Data are from two independent experiments. In **A**, experiment 1: mouse, n = 3; experiment 2: mouse, n = 4. In **B**, experiment 1: rat, n = 3; experiment 2: rat, n = 3.
(TIF)

S5 Fig Number of worms recovered from WT and *Lck*^*−/−*^ rat at 6-week infection. Data are from two independent experiments. Experiment 1: WT, n = 3; *Lck*^*−/−*^, n = 6. Experiment 2: WT, n = 3; *Lck*^*−/−*^, n = 7.
(TIF)

S6 Fig Generation and analysis of *CD3e* knock-out SD rat. (A) Schematic diagram of the rat *CD3e* locus and sgRNA targeting sequences. sgRNA 1 and sgRNA 2 are in exon 4; sgRNA 3 and sgRNA 4 are in exon 5. (B) Representative FACS analysis of T cells and cell number of total T cells, CD4^+^ and CD8^+^ T cells in peripheral blood of WT and *CD3e*^*−/−*^ rat (WT, n = 6; *CD3e*^*−/−*^, n = 5). (C) Representative FACS analysis of T cells and cell count of total T cells, CD4^+^ and CD8^+^ T cells in spleen of WT and *CD3e*^*−/−*^ rat (WT, n = 6; *CD3e*^*−/−*^, n = 5). The following fluorochrome-tagged antibodies were used: anti-Rat CD45-eFluor450, anti-Rat CD3-FITC, anti-Rat CD4-APC/Cyanine7. Data represent the mean ± s.e.m. Statistical significance was assessed by unpaired Student’s *t*-test or non-parametric unpaired Mann-Whitney test and indicated by ** *P*<0.01, *** *P*<0.001.
(TIF)

S7 Fig Pathologic analysis of WT and *CD3e*^*−/−*^ rat after *S*. *japonicum* infection. (A) Number of male and female worms recovered from WT and *CD3e*^***−/−***^ SD rat at 6-week post-infection. (B) Number of worms recovered from WT and *CD3e*^*−/−*^ rat at 6-week post-infection. (C) The recovery rate of *S*. *japonicum* collected from infected WT rat and *CD3e*^***−/−***^ rat. Data are from one experiment (WT, n = 4; *CD3e*^*−/−*^, n = 4). (D) H&E and Masson’s trichrome staining of liver sections from uninfected or infected WT and infected *CD3e*^−/−^ rat. (Original magnification, ×100; Scale bar, 100 μm.). (E) Granuloma size measured from H&E staining of liver sections using CaseViewer software (WT, n = 6; *CD3e*^*−/−*^, n = 5). (F) Fibrotic areas measured from Masson’s trichrome staining of liver sections using CaseViewer software (WT, n = 6; *CD3e*^*−/−*^, n = 5). (G) qPCR analysis of mRNA expression of fibrosis-related genes in liver tissue from uninfected or infected WT and infected *CD3e*^−/−^ rat: *Col1α1*, *Col3α1 and α-SMA*. (H) CD11b (left) and CD19 (right) staining of liver sections from uninfected or infected WT rat and infected *CD3e*^−/−^ rat. (Original magnification, ×100; Scale bar, 100 μm). (I) CD11b^+^ (left) and CD19^+^ (right) percentage measured from CD11b and CD19 staining of liver sections of infected WT rat in comparison of *CD3e*^−/−^ rat using CaseViewer software (WT, n = 5; *CD3e*^*−/−*^, n = 5) (right). The reference data showing pathology of WT rat in **E**, **F** and **I** were used in Figs 4 and 5. Statistical significance was assessed by unpaired Student’s *t*-test or non-parametric unpaired Mann-Whitney test and indicated by * *P*<0.05, ** *P*<0.01, *** *P*<0.001, **** *P*<0.0001.
(TIF)

S8 Fig Antibody level in T cell deficient mice and rats following *S*. *japonicum* infection. The level of IgG anti-SWAP in the sera of WT mouse or *CD3e*^*−/−*^ mouse (A) and WT rat or *Lck*^***−/−***^ rat (B) detected using ELISA. Data represent the mean ± s.e.m.
(TIF)

S1 Table Primers for real-time quantitative PCR. (DOCX)
