# Supplementary figures and images for: Loss of natural resistance to schistosome in T cell deficient rat
Source: PLoS Negl Trop Dis. 2020 Dec 21;14(12):e0008909. doi: 10.1371/journal.pntd.0008909 (PMC7785244; doi:10.1371/journal.pntd.0008909)

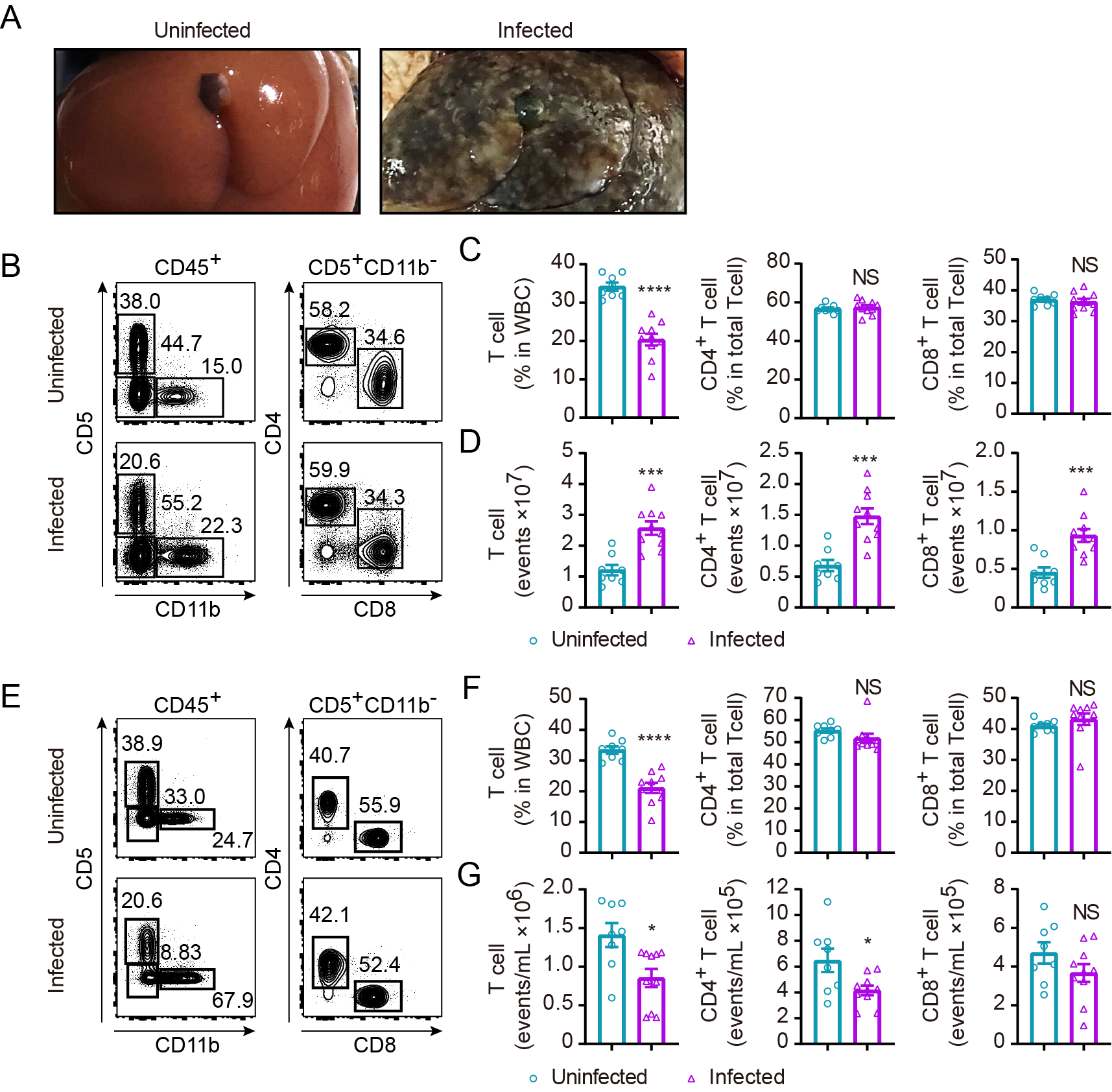

Supplement: S1 Fig — (A) liver granuloma of B6 mice 6 weeks after infection with 20 cercariae of S. japonicum, uninfected (left), infected (right). (B) Multiparameter FACS analysis of splenocytes in B6 mice under uninfected and infected conditions, identifying T cells (CD5+) and myeloid cells (CD11b+) among CD45+ white blood cells (left), and further subcategorizing CD5+ T cells into CD4+ or CD8+ cells (right). Numbers adjacent to outlined areas indicate percent cells in each gate. (C) Quantification of the data in (B). (D) Absolute number of total T cells, CD4+ or CD8+ T cells in spleen from uninfected and schistosome infected B6 mice. Results from two independent experiments (uninfected, n = 8; infected, n = 10). (E) Representative FACS analysis of white blood cells and T cells in peripheral blood of uninfected B6 or 6-week infected mice. (F, G) Percentages and absolute number of total T cells, CD4+ or CD8+ T cells in E. Results represented for two independent experiments (uninfected, n = 8; infected, n = 10). The following fluorochrome-tagged antibodies were used: anti-mouse CD45-APC-eFluor 780, anti-mouse CD5-PE-Cyanine7, anti-mouse CD11b APC, anti-mouse CD4-FITC, anti-mouse CD8α-Alexa Fluor 700. Data represent the mean ± s.e.m. Statistical significance was assessed by unpaired Student’s t-test or non-parametric unpaired Mann-Whitney test and indicated by * P<0.05, *** P<0.001, **** P<0.0001, NS, non-significant. (TIF) [file pntd.0008909.s001.tif]

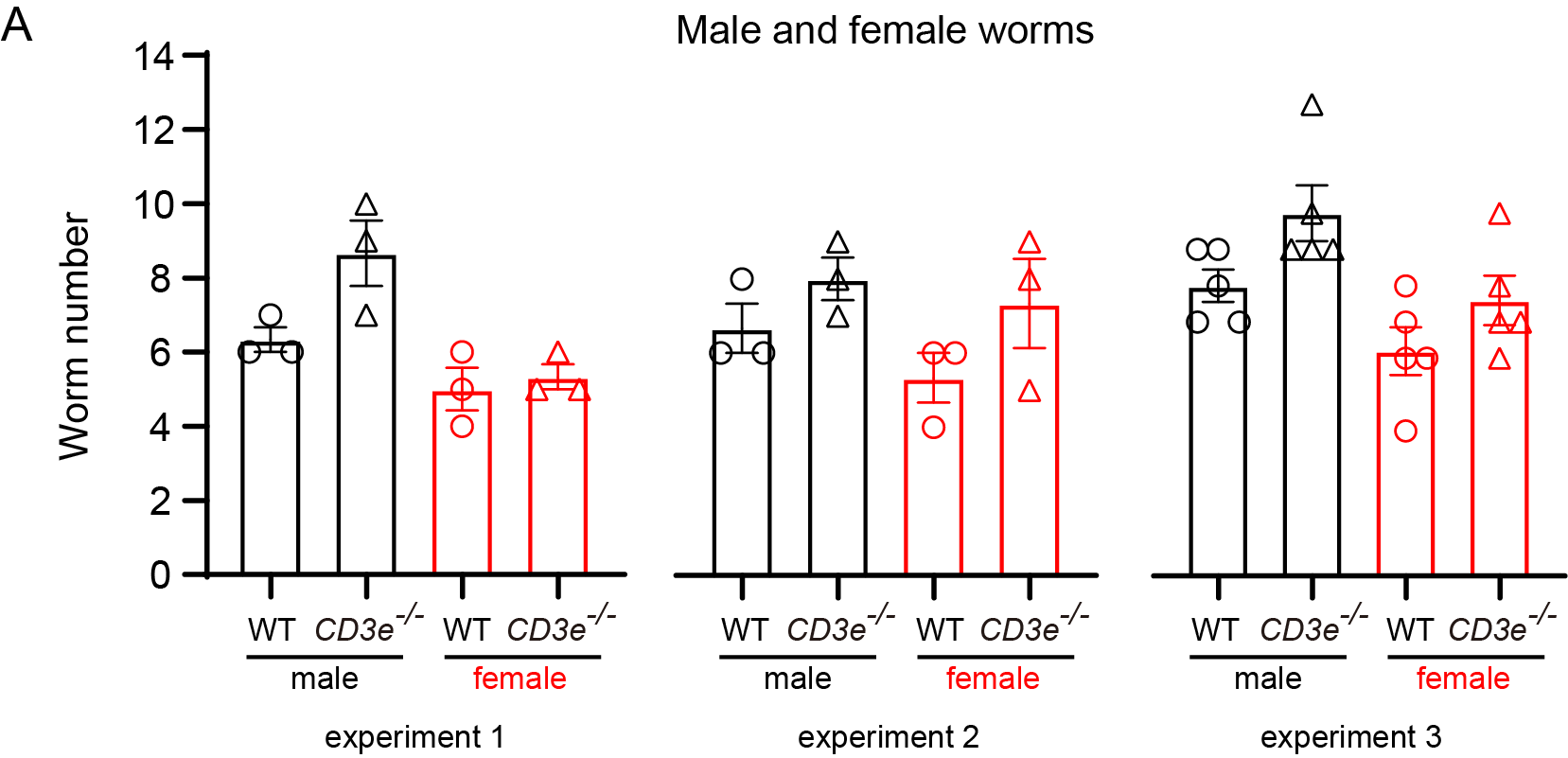

Supplement: S2 Fig — Data are from three independent experiments (Experiment 1: WT, n = 3; CD3e−/−, n = 3. Experiment 2: WT, n = 3; CD3e−/−, n = 3. Experiment 3: WT, n = 5; CD3e−/−, n = 5). (TIF) [file pntd.0008909.s002.tif]

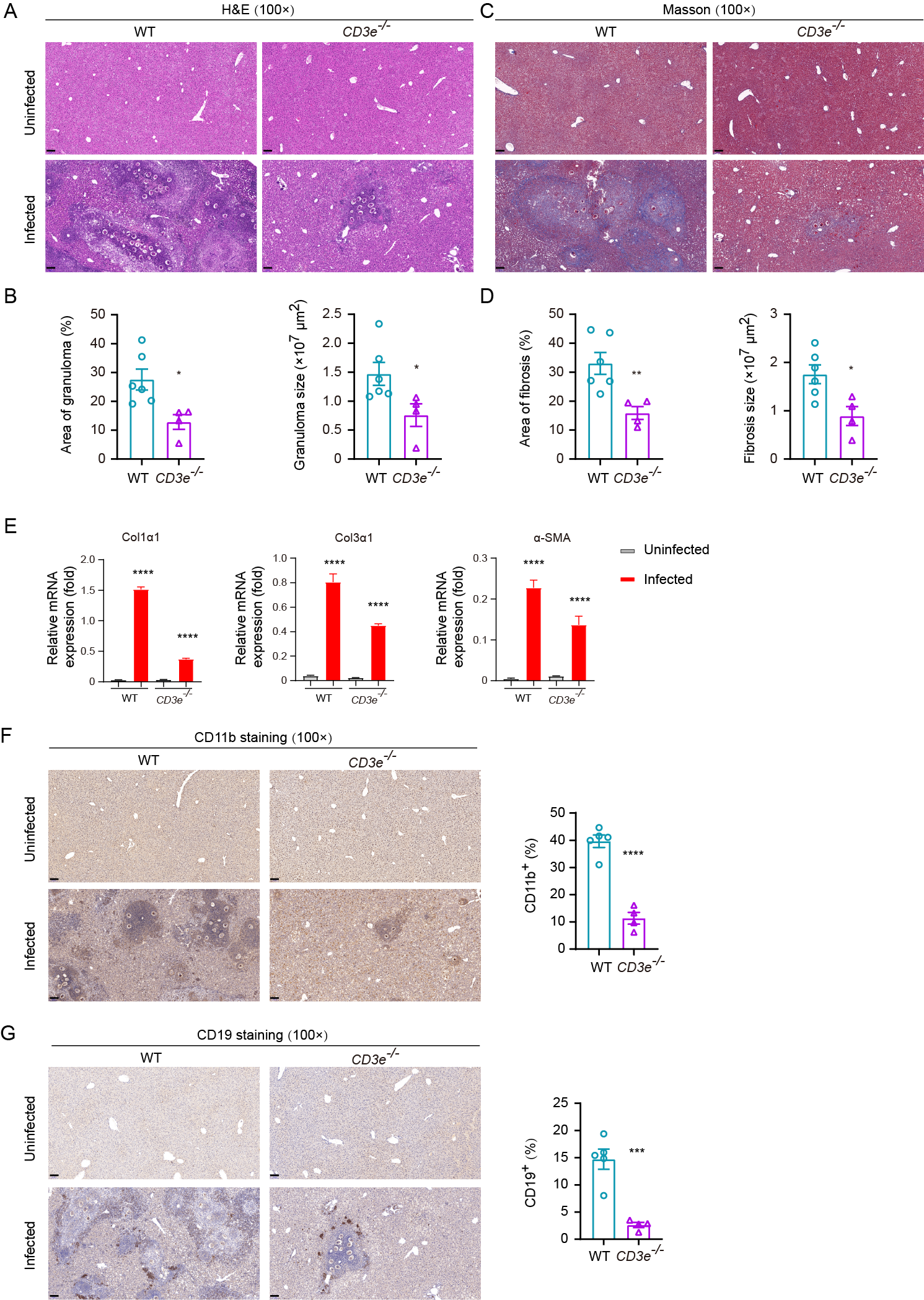

Supplement: S3 Fig — (A) H&E staining of liver sections from uninfected or infected WT B6 and CD3e−/− mouse. (Original magnification, ×100; Scale bar, 100 μm.). (B) Granuloma size was quantified from H&E stained liver sections using CaseViewer software (WT, n = 6; CD3e−/−, n = 4). (C) Masson’s trichrome staining of liver sections from uninfected or infected WT B6 and CD3e−/− mouse. (Original magnification, ×100; Scale bar, 100 μm.). (D) Fibrotic areas from Masson’s trichrome staining of liver sections by using CaseViewer software (WT, n = 6; CD3e−/−, n = 4). (E) qPCR analysis of mRNA expression level of fibrosis-related genes: Col1α1, Col3α1 and α-SMA in the liver tissues from uninfected or infected WT B6 and CD3e−/− mouse. (F) CD11b staining of liver sections from infected or uninfected WT B6 and CD3e−/− mouse. (Original magnification, ×100; Scale bar, 100 μm.) (left). CD11b+ area shown in percentage measured from CD11b stained liver sections by using CaseViewer software (WT, n = 5; CD3e−/−, n = 4) (right). (G) CD19 staining of liver sections from infected or uninfected WT B6 and CD3e−/− mouse. (Original magnification, ×100; Scale bar, 100 μm.) (left). CD19+ area shown in percentage measured from CD19 staining of liver sections by using CaseViewer software (WT, n = 5; CD3e−/−, n = 4) (right). Data represent the mean ± s.e.m. Statistical significance was assessed by unpaired Student’s t-test or non-parametric unpaired Mann-Whitney test and indicated by * P<0.05, **** P<0.0001, NS, non-significant. (TIF) [file pntd.0008909.s003.tif]

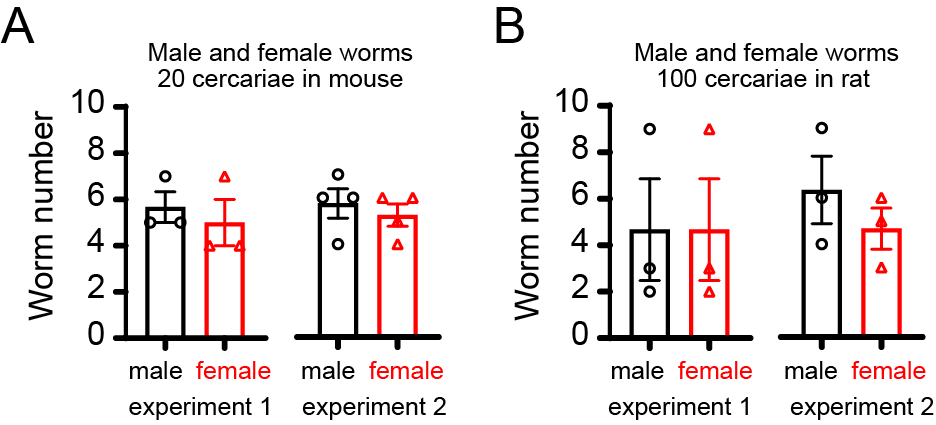

Supplement: S4 Fig — Numbers of worms from WT B6 mouse (A) and WT SD rat (B) at 6-week post-challenge. Data are from two independent experiments. In A, experiment 1: mouse, n = 3; experiment 2: mouse, n = 4. In B, experiment 1: rat, n = 3; experiment 2: rat, n = 3. (TIF) [file pntd.0008909.s004.tif]

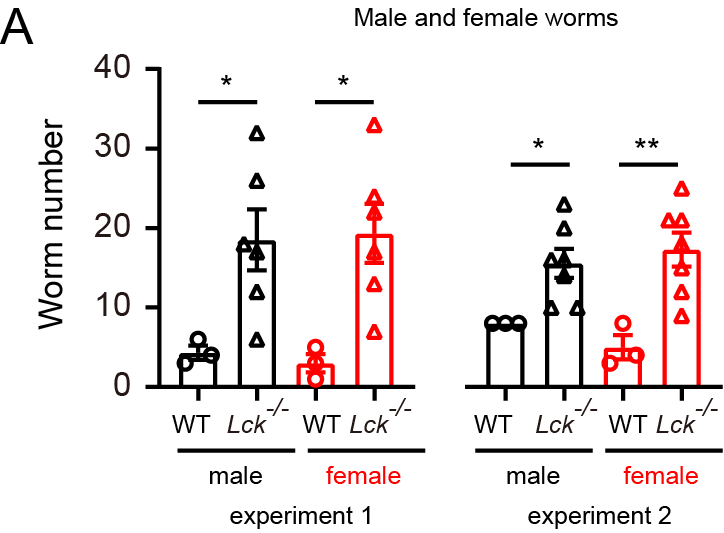

Supplement: S5 Fig — Data are from two independent experiments. Experiment 1: WT, n = 3; Lck−/−, n = 6. Experiment 2: WT, n = 3; Lck−/−, n = 7. (TIF) [file pntd.0008909.s005.tif]

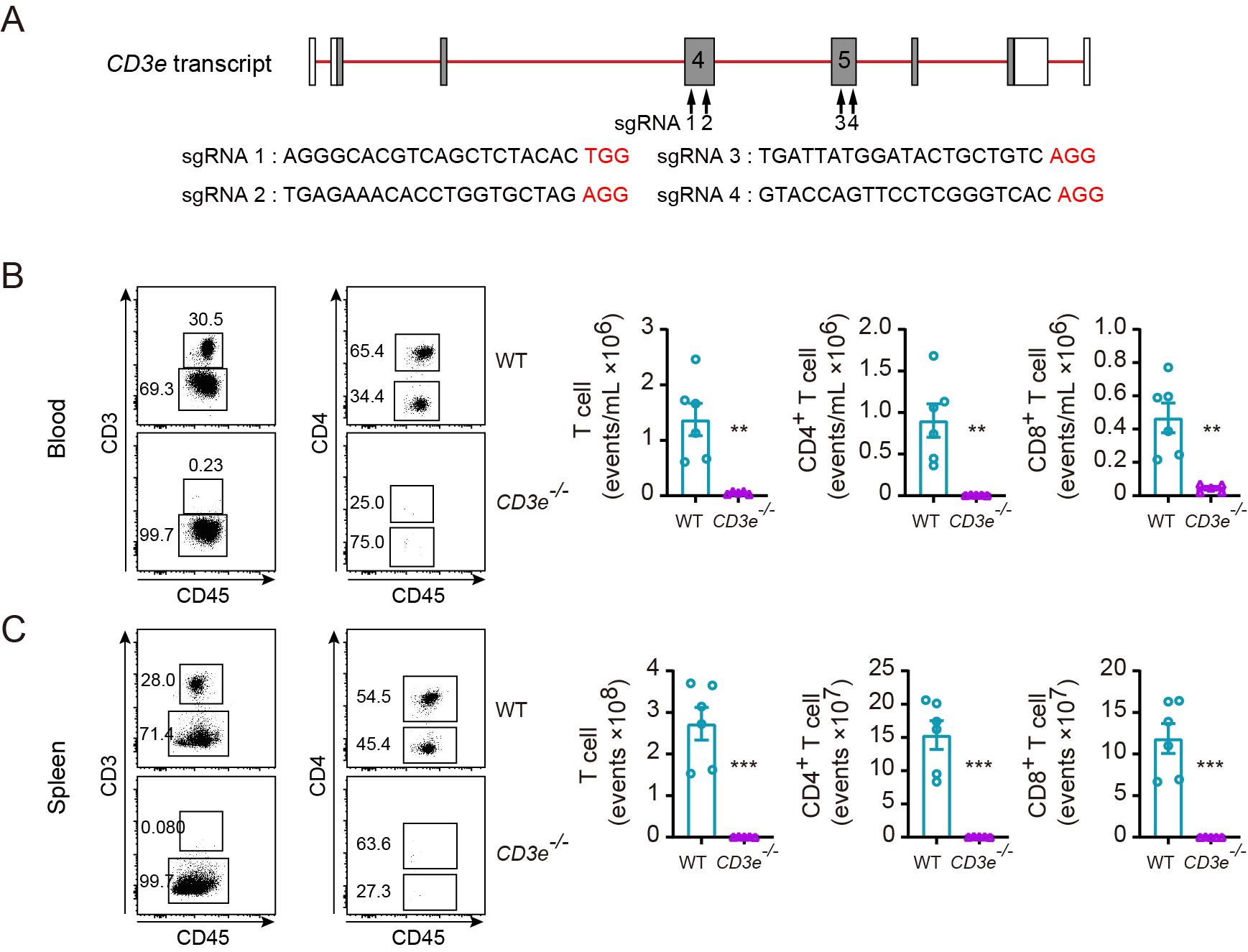

Supplement: S6 Fig — (A) Schematic diagram of the rat CD3e locus and sgRNA targeting sequences. sgRNA 1 and sgRNA 2 are in exon 4; sgRNA 3 and sgRNA 4 are in exon 5. (B) Representative FACS analysis of T cells and cell number of total T cells, CD4+ and CD8+ T cells in peripheral blood of WT and CD3e−/− rat (WT, n = 6; CD3e−/−, n = 5). (C) Representative FACS analysis of T cells and cell count of total T cells, CD4+ and CD8+ T cells in spleen of WT and CD3e−/− rat (WT, n = 6; CD3e−/−, n = 5). The following fluorochrome-tagged antibodies were used: anti-Rat CD45-eFluor450, anti-Rat CD3-FITC, anti-Rat CD4-APC/Cyanine7. Data represent the mean ± s.e.m. Statistical significance was assessed by unpaired Student’s t-test or non-parametric unpaired Mann-Whitney test and indicated by ** P<0.01, *** P<0.001. (TIF) [file pntd.0008909.s006.tif]

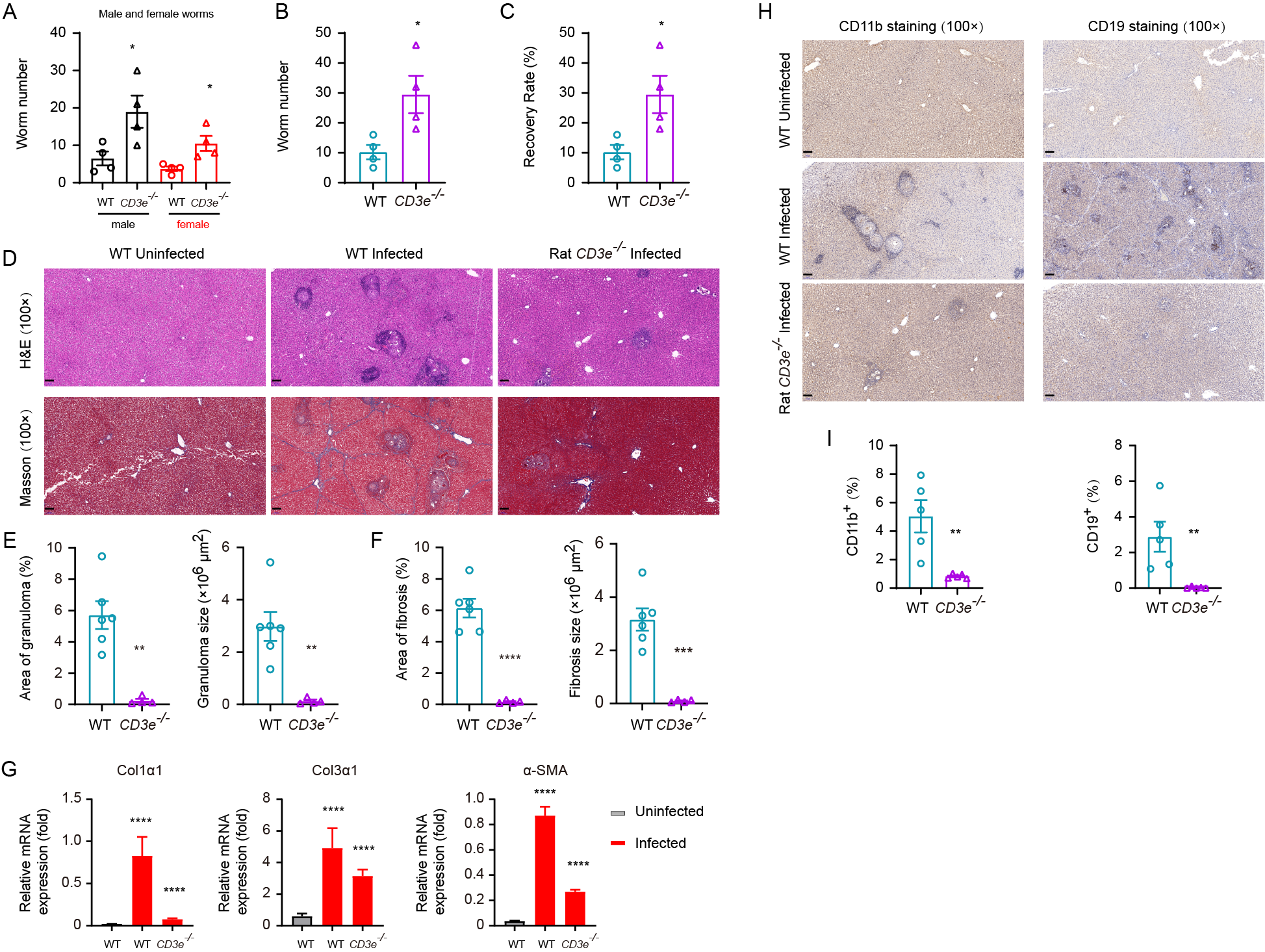

Supplement: S7 Fig — (A) Number of male and female worms recovered from WT and CD3e−/− SD rat at 6-week post-infection. (B) Number of worms recovered from WT and CD3e−/− rat at 6-week post-infection. (C) The recovery rate of S. japonicum collected from infected WT rat and CD3e−/− rat. Data are from one experiment (WT, n = 4; CD3e−/−, n = 4). (D) H&E and Masson’s trichrome staining of liver sections from uninfected or infected WT and infected CD3e−/− rat. (Original magnification, ×100; Scale bar, 100 μm.). (E) Granuloma size measured from H&E staining of liver sections using CaseViewer software (WT, n = 6; CD3e−/−, n = 5). (F) Fibrotic areas measured from Masson’s trichrome staining of liver sections using CaseViewer software (WT, n = 6; CD3e−/−, n = 5). (G) qPCR analysis of mRNA expression of fibrosis-related genes in liver tissue from uninfected or infected WT and infected CD3e−/− rat: Col1α1, Col3α1 and α-SMA. (H) CD11b (left) and CD19 (right) staining of liver sections from uninfected or infected WT rat and infected CD3e−/− rat. (Original magnification, ×100; Scale bar, 100 μm). (I) CD11b+ (left) and CD19+ (right) percentage measured from CD11b and CD19 staining of liver sections of infected WT rat in comparison of CD3e−/− rat using CaseViewer software (WT, n = 5; CD3e−/−, n = 5) (right). The reference data showing pathology of WT rat in E, F and I were used in Figs 4 and 5. Statistical significance was assessed by unpaired Student’s t-test or non-parametric unpaired Mann-Whitney test and indicated by * P<0.05, ** P<0.01, *** P<0.001, **** P<0.0001. (TIF) [file pntd.0008909.s007.tif]

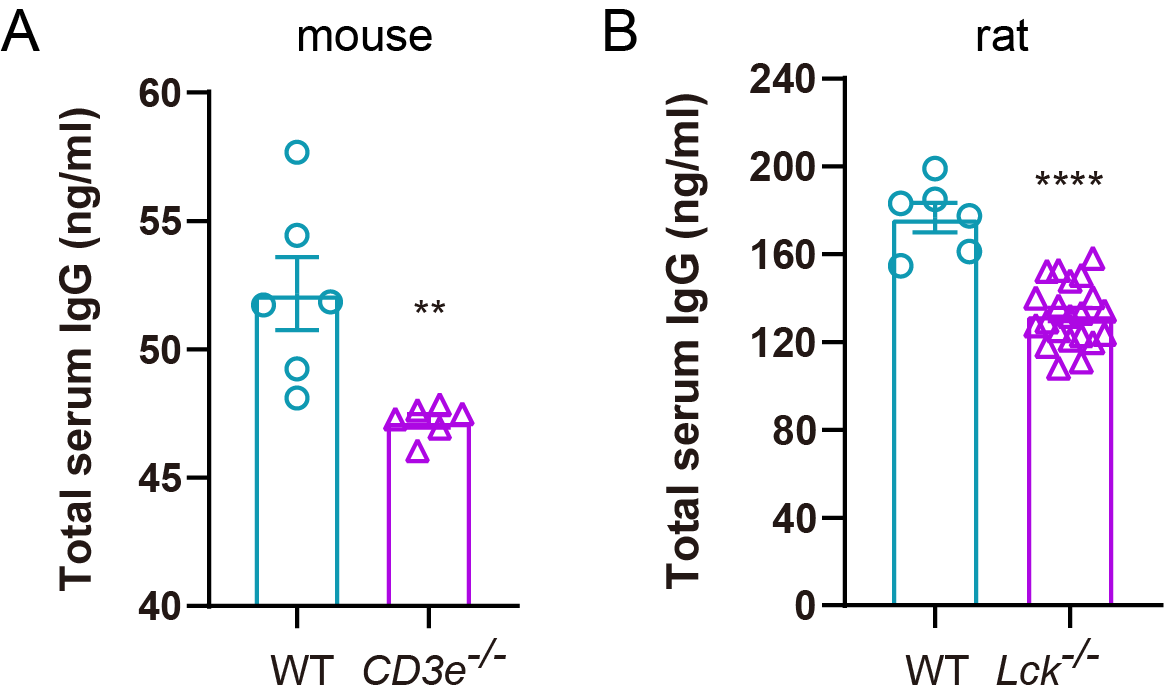

Supplement: S8 Fig — The level of IgG anti-SWAP in the sera of WT mouse or CD3e−/− mouse (A) and WT rat or Lck−/− rat (B) detected using ELISA. Data represent the mean ± s.e.m. (TIF) [file pntd.0008909.s008.tif]
