# Supplementary material for: Loss of natural resistance to schistosome in T cell deficient rat
Source: PLoS Negl Trop Dis. 2020 Dec 21;14(12):e0008909. doi: 10.1371/journal.pntd.0008909 (PMC7785244; doi:10.1371/journal.pntd.0008909)
Supplement: S1 Table — (DOCX) [file pntd.0008909.s009.docx]

**S1 Table.** **Primers for real-time quantitative PCR.**

| **Animal** | **Primer name** | **Primer sequence (5’ > 3’)** | **Amplicon length** |
| --- | --- | --- | --- |
| rat | r-Col1α1-F | AGCATGTCTGGTTTGGAGAGAG | 141 bp |
|  | r-Col1α1-R | GATAGGTGATGTTCTGGGAGGC |  |
|  | r-Col3α1-F | AGACCTGAAATTCTGCCACCC | 111 bp |
|  | r-Col3α1-R | TCTCCGGTTTCCATATTACAGAACA |  |
|  | r-α-SMA-F | AGATCTCACCGACTACCTCATGA | 157 bp |
|  | r-α-SMA-R | GGAAGAAGAGGAAGCAGCAGT |  |
|  | r-Sdha-F | GCTTTAACACGGCATGCCTT | 149 bp |
|  | r-Sdha-R | CAGCCAGTCAGAGCCTTTCA |  |
|  | r-Tbx21-F | CCGCTTATACGTCCACCCAG | 160 bp |
|  | r-Tbx21-R | CGGCTGGTACTTATGGAGGG |  |
|  | r-Gata3-2F | GGGCTGTACTACAAACTCCACA | 158 bp |
|  | r-Gata3-2R | TTGAAGGAGCTGCTCTTGGG |  |
|  | r-Rorc-2F | CACCAACCTCTTCTCACGGG | 153 bp |
|  | r-Rorc-2R | CACAGCTCCATGAAGCCTGA |  |
|  | r-Bcl6-1F | CCGGAAGTTCATCAAGGCCA | 136 bp |
|  | r-Bcl6-1R | CTCCACAACCTCACGACCTC |  |
| mouse | m-Col1α1-F | GCACGAGTCACACCGGAAC | 100 bp |
|  | m-Col1α1-R | CCAATGTCCAAGGGAGCCAC |  |
|  | m-Col3α1-F | AAGCCAGAACCATGTCAAATATGTG | 156 bp |
|  | m-Col3α1-R | CACAGGAGCAGGTGTAGAAGG |  |
|  | m-α-SMA-F | GTCCCAGACATCAGGGAGTAA | 102 bp |
|  | m-α-SMA-R | TCGGATACTTCAGCGTCAGGA |  |
|  | m-Hprt-F | CGTCGTGATTAGCGATGATG | 184 bp |
|  | m-Hprt-R | ACAGAGGGCCACAATGTGAT |  |
|  | m-Tbx21-F | AGCTCACCAACAACAAGGGG | 144 bp |
|  | m-Tbx21-R | TGTTAGAAGCACTGCAGGCA |  |
|  | m-Gata3-F | GACGGAAGAGGTGGACGTAC | 160 bp |
|  | m-Gata3-R | CAGAGATCCGTGCAGCAGAG |  |
|  | m-Rorc-F | CCTTGCAAGATCTGTGGGGA | 122 bp |
|  | m-Rorc-R | GTGCAGGAGTAGGCCACATT |  |
|  | m-Bcl6-1F | CGTGAGCAGTTTAGAGCCCA | 150 bp |
|  | m-Bcl6-1R | CAGGAGGATGCAAAACCCCT |  |
